# At the Edge of Survival: Exploring the Frontiers of Tardigrade Extreme Stress Tolerance

**DOI:** 10.1111/mec.70471

**Published:** 2026-07-22

**Authors:** Maria Kamilari, Frida Løkkegaard Pust, Andrea Marcantognini, Ask Møbjerg, Ricardo Cardoso Neves, Morten Schiøtt, Nadja Møbjerg

**Affiliations:** ^1^ Department of Plant Protection Patras, Institute of Industrial and Forage Crops Hellenic Agricultural Organization ‘DIMITRA’ Patras Greece; ^2^ Department of Biology University of Copenhagen Copenhagen Denmark; ^3^ Department of Biotechnology and Biomedicine Technical University of Denmark Kgs. Lyngby Denmark

**Keywords:** cryptobiosis, data mining, differential gene expression, genomics, molecular mechanisms, NGS, RNA‐seq, tardigrades, transcriptomics

## Abstract

Tardigrades possess extraordinary resilience to extreme environmental conditions, including near‐complete desiccation, severe levels of radiation, the vacuum of space and extreme sub‐zero temperatures. Despite significant interest in their survival strategies, the molecular mechanisms underlying their survival during extreme stresses have remained elusive. However, recent sequencing approaches and gene expression analyses have significantly expanded our knowledge of tardigrade biology. This review explores how omics technologies, particularly RNA sequencing and other RNA‐based methods are currently transforming our understanding of tardigrade survival strategies, identifying genes with proposed significance for tardigrade extreme stress tolerance. We briefly discuss characteristics of tardigrade species with available transcriptome and genome assemblies and, by synthesising insights from recent studies, provide a thorough examination of proposed mechanisms involved in tardigrade resilience. We share our curated data‐mining results and also provide a new transcriptome for the species *Echiniscus testudo* . Proposed mechanisms are addressed from a whole phylum perspective, emphasising evolutionary relationships and the proposed origin of identified genes. These genes encode proteins involved in mechanisms such as antioxidant defence, DNA protection and repair, protein folding and protection of three‐dimensional structure from molecular and cellular to whole animal levels. Special focus is on peroxins and also filament‐forming proteins, which likely play a vital role in sustaining the so‐called tun state—a state that enables desiccated tardigrades to suspend metabolism, yet uphold structural integrity for decades before returning to life following resuspension in water. Finally, we discuss promising future directions for using omics technologies and RNA‐based methods in unravelling tardigrade survival strategies.

**Abbreviations**
AMNP: tardigrade‐specific Mn‐peroxidase
AQP: aquaporin
ATHL1: acid trehalase‐like 1
BCS1: cytochrome BC1 synthesis 1
BER: base excision repair
CAHS: cytoplasmic abundant heat soluble
Cas: CRISPR‐associated system
Cas9: CRISPR‐associated protein 9
CAT: catalase
cDNA: complementary deoxyribonucleic acid
CRISPR: clustered regularly interspaced short palindromic repeats
CSD: cold shock domain
CSP: cold shock protein
DNA: deoxyribonucleic acid
DODA1: DOPA dioxygenase 1
dsRNA: double‐stranded ribonucleic acid
Dsup: damage suppressor
EST: expression sequence tag
EtAHS:
*Echiniscus testudo* abundant heat soluble
FABP: fatty acid binding protein
GO: gene ontology
GPX: glutathione peroxidase
GSS: glutathione synthetase
HGT: horizontal gene transfer
HMM: hidden Markov model
HR: homologous recombination
HSP: heat shock protein
IDPs: intrinsically disordered proteins
IR: ionising radiation
KEGG: Kyoto Encyclopedia of Genes and Genomes
KOG: eukaryotic orthologous groups
LEA: late embryogenesis abundant
Lin28: abnormal cell lineage family member 28
MAHS: mitochondrial abundant heat soluble
miRNA: micro ribonucleic acid
MPV17: MPV17 mitochondrial inner membrane protein
MRE11: meiotic recombination protein 11
mRNA: messenger ribonucleic acid
mRNA‐seq: mRNA sequencing
ncRNA: non‐coding ribonucleic acid
NDUFB8: NADH dehydrogenase [ubiquinone] 1 beta subcomplex subunit
NGS: next generation sequencing
NHEJ: non‐homologous end joining
NR: non‐redundant protein database
NT: nucleotide database
p53: p53 superfamily involved in DNA repair
PEX: peroxin
PRDX: peroxiredoxin
PTS1: peroxisomal targeting signal type 1
PTS2: peroxisomal targeting signal type 2
PXMP/PMP: peroxisomal membrane proteins
Rad51: radiation sensitive 51
RNA: ribonucleic acid
RNAi: RNA interference
RNA‐seq: RNA sequencing
ROS: reactive oxygen species
rRNA: ribosomal ribonucleic acid
RvLEAM:
*Ramazzottius varieornatus* late embryogenesis abundant mitochondrial
SAHS: secretory abundant heat soluble
scRNA‐seq: single‐cell RNA sequencing
sgRNA: single guide RNA
sHSP: small heat shock protein
siRNA: short interfering ribonucleic acid
SOD: superoxide dismutase
TDR1: tardigrade DNA damage response protein 1
TPP: trehalose‐6‐phosphate phosphatase
TPS: trehalose‐6‐phosphate synthase
TRID1: tardigrade‐specific radiation induced disordered protein 1
tRNA: transfer ribonucleic acid
TXN: thioredoxin
TXNL: thioredoxin‐like
TXNRD: thioredoxin Reductase
TYSND1: trypsin domain containing 1
XRCC: X‐ray repair cross‐complementing
YB: Y‐box

## 1 Introduction

### 1.1 Tardigrades

**FIGURE 1:**
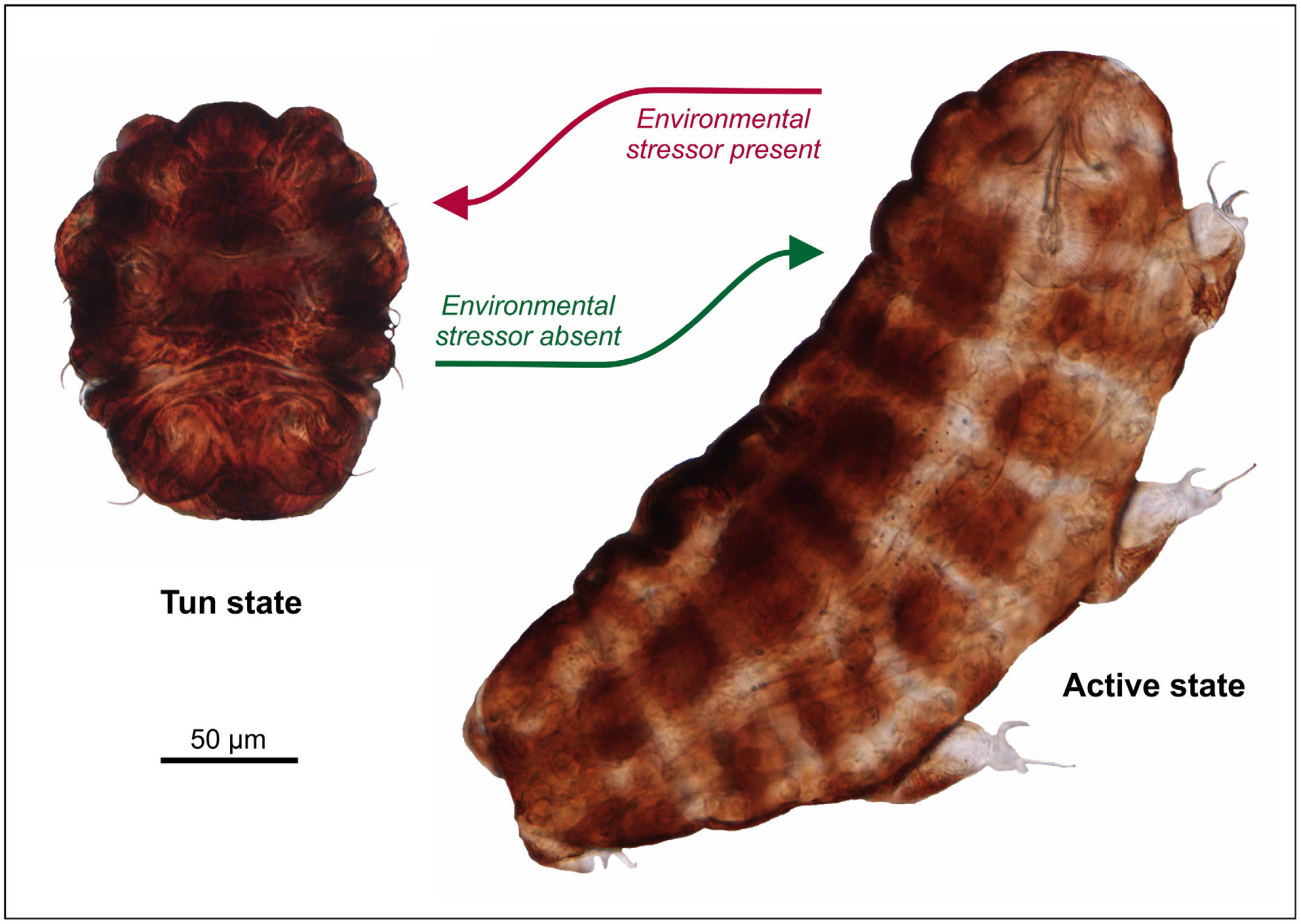
Cryptobiosis in tardigrades. Light micrographs of the limno‐terrestrial eutardigrade *Ramazzottius varieornatus* in its active and tun state, respectively. During exposure to environmental stressors, such as desiccation or high osmotic pressure, the tardigrade will withdraw its head and legs and contract its body, thereby forming a quiescent tun. Upon return to freshwater, the tardigrade tun takes up water, extends its body and once again returns to its active state.

Tardigrades (Figure 1), also known as ‘water bears’ or ‘moss piglets’, have received great attention due to their exceptional resilience to extreme environmental conditions, which would prove fatal for most other organisms (Møbjerg et al. 2011; Erdmann and Kaczmarek 2017; Hibshman et al. 2020; Rolland et al. 2024). These microscopic eight‐legged aquatic organisms (Møbjerg et al. 2018) exhibit extraordinary survival abilities, enduring near complete desiccation (Sømme 1996; Rebecchi et al. 2007; Halberg et al. 2013; Wełnicz et al. 2011; Arakawa 2022), severe levels of radiation (Horikawa et al. 2006; Jönsson 2019; Anoud et al. 2024), the vacuum of space (Jönsson et al. 2008; Persson et al. 2011) and extreme sub‐zero temperatures (Hengherr et al. 2009; Møbjerg et al. 2022; Lee et al. 2022). It is generally accepted that extreme stress tolerance in tardigrades involves the ability to enter cryptobiosis—a state of metabolic arrest (Clegg 2001; Wright 2001; Neuman 2006; Møbjerg and Neves 2021) which allows tardigrades to survive otherwise lethal conditions until favourable settings return. Cryptobiosis exists in several sub‐states, induced by different abiotic factors such as osmobiosis (high osmotic pressure), anhydrobiosis (desiccation), cryobiosis (freezing) and chemobiosis (environmental toxicants), involving formation of a so‐called ‘tun’ (Møbjerg and Neves 2021; Hvidepil and Møbjerg 2023; Smythers et al. 2024). During tun formation, tardigrades contract their body significantly, while withdrawing their head and legs (Figure 1).

Although the remarkable adaptations of tardigrades have attracted extensive scientific attention, the mechanisms enabling their survival during extreme stresses have remained a mystery for decades. While early investigations into tardigrade biology provided glimpses into their unique survival strategies (Kinchin 1994; Greven 2018), the emergence of high‐throughput sequencing technologies has revolutionised our ability to investigate the genetic and molecular basis of tardigrade resilience (Hashimoto et al. 2016; Yoshida et al. 2017; Kamilari et al. 2019; Clark‐Hachtel et al. 2024; Anoud et al. 2024; Fleming et al. 2024; Li et al. 2024). This review explores how new sequencing technologies, and especially RNA‐based approaches, are transforming our understanding of tardigrade biology, shedding new light on mechanisms underlying the ability of tardigrades to withstand extreme stressors. By synthesising insights from recent sequencing and experimental studies, we provide an overview of the current state of knowledge and highlight future directions for tardigrade research.

### 1.2 RNAs and Transcriptomics

During transcription of DNA, a complementary RNA molecule is synthesised—these transcripts serve as a link between DNA and protein, but also as key regulators of cellular processes. Hence, RNA is dynamic and diverse, involving both coding RNA and non‐coding RNA (ncRNA), with each type of RNA fulfilling distinct roles. RNA involved in protein synthesis includes protein‐coding messenger RNA (mRNA) molecules, as well as ribosomal RNA (rRNA) and transfer RNA (tRNA). Ribosomal RNAs account for 80%–90% of the cell's total RNA mass (Palazzo and Lee 2015), and the highly conserved regions of rRNA genes are extensively used in phylogenetic studies of organisms across life kingdoms. Accordingly, both 18S and 28S rRNA are widely used in tardigrade phylogenetics (Garey et al. 1996; Sands et al. 2008; Jørgensen et al. 2010; Guil and Giribet 2012; Bertolani et al. 2014; Guil et al. 2018; Morek and Michalczyk 2019; Stec et al. 2021; Vecchi et al. 2023; Erözden et al. 2024). Moreover, 18S rRNA has recently been used in metabarcoding studies on tardigrades (Topstad et al. 2021; Pust et al. 2024; He et al. 2024).

**FIGURE 2:**
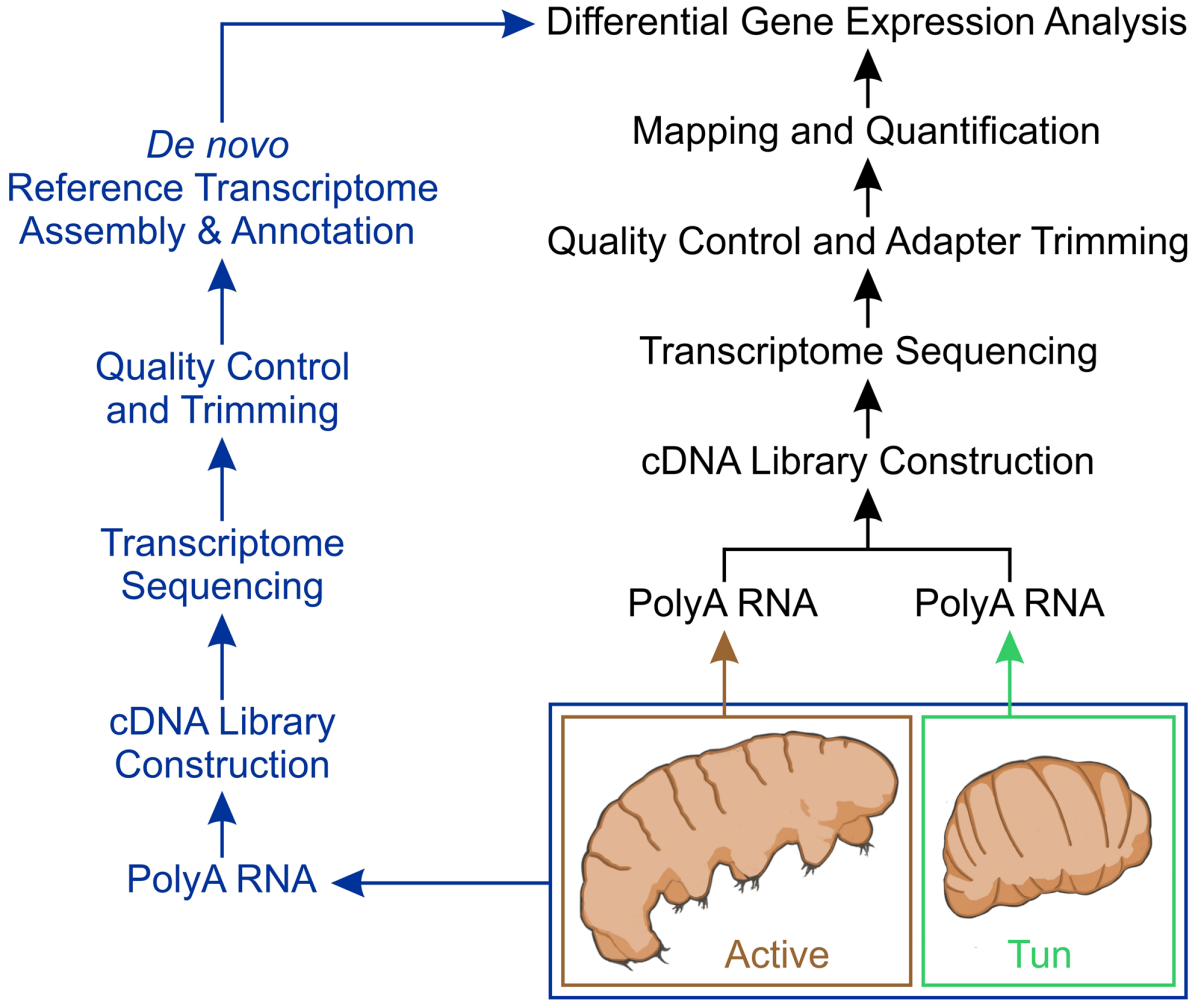
Overview of workflows involved in mRNA‐seq analyses. Schematic overview of mRNA selection, RNA‐seq and bioinformatic workflows involved in differential gene expression analyses, aiming to compare expression profiles between, for example, active and tun state tardigrades. Following total RNA purification, mRNA is poly‐A tail selected, reverse transcribed and cloned into a suitable vector for cDNA library preparation and subsequent sequencing on NGS or, more recently, third‐generation long read platforms. Sequenced reads (black lines and arrows) are mapped against a genome, or a reference transcriptome (blue workflow) assembled de novo from a pool of tardigrades representing different developmental and physiological stages. Raw reads are subjected to quality control and trimming of, for example, adapters and the obtained clean reads are subsequently quantified, after which genes with too many missing counts across biological replicates may be removed. A wide array of computational methods for analysing differential gene expression of RNA‐seq data have been developed, each potentially introducing different biases in the identification of these genes. Commonly used bioconductor packages include DESeq2 and EdgeR.

Transcriptomics, the study of all RNA expressed by a genome at a given time, offers a powerful tool to unravel complex molecular mechanisms underlying adaptation. Early transcriptomic studies relied on low‐throughput Sanger sequencing of random transcripts, producing expressed sequence tags (ESTs), that is, short fragments of mRNA sequences generated by single‐pass sequencing of randomly selected clones from complementary DNA (cDNA) libraries (Parkinson and Blaxter 2009). While this method was limited by its low throughput, it nonetheless allowed the first identification of genes expressed under stress conditions in tardigrades, including *Milnesium inceptum* (Mali et al. 2010). More recently, RNA sequencing (RNA‐seq) has revolutionised research into molecular mechanisms underlying tardigrade stress tolerance by relying on high‐throughput next‐generation sequencing (NGS) of cDNA (Nagalakshmi et al. 2010; Wang et al. 2009). RNA‐seq enables the identification and quantification of all RNA transcripts, including both mRNA and ncRNA and offers several advantages over other methods, such as higher sensitivity, wider dynamic range and the ability to detect novel transcripts and alternative splicing events (Goldstein et al. 2016; Weirick et al. 2016). This technique enables comparative transcriptome investigations across tardigrade species as well as detailed investigations into differential gene expression in specimens of the same species subjected to different environmental conditions. Importantly, differential gene expression analyses require a reference genome or transcriptome, substantial computational resources and expertise for data analyses and interpretation (Figure 2). In addition, as RNA‐seq currently builds on short‐read NGS technology and relies on copying RNA into cDNA, errors and biases related to short sequences and reverse transcription are likely to be introduced, leading to challenges in gene prediction (Engström et al. 2013; Vijay et al. 2013; Venket Raghavan et al. 2022). These challenges will likely be significantly remedied by more recently developed long‐read sequencing technologies. Specifically, direct sequencing of RNA using Nanopore sequencing technology can enhance the quality of RNA sequencing, avoiding the errors and biases related to reverse transcription and short sequences.

#### 1.2.1 Non‐Coding RNAs

RNA‐mediated mechanisms generally play a critical role in gene regulation, and can therefore be used as powerful tools in investigations of developmental and physiological processes (Santosh et al. 2014; Huang and Zhang 2014). Although great focus in recent years has been on tardigrade RNA sequencing, knowledge of the role of tardigrade ncRNAs, including, for example, micro‐RNA (miRNA) and short interfering RNA (siRNA) is still sparse. Specifically, small RNAs, including small ca. 22‐nucleotide miRNAs and siRNAs (Bartel 2004), can regulate gene expression by targeting mRNAs for degradation or inhibiting their translation. By binding to matching mRNA transcripts, miRNA destabilise mRNA by various mechanisms, thereby reducing protein synthesis (Guo et al. 2010). Interestingly, miRNAs are continually being added to bilaterian lineages but are apparently rarely lost (Wheeler et al. 2009). Also, miRNAs show a high degree of sequence conservation as their function is dependent on perfect alignment to their gene target, and mutational changes will therefore be selected against unless the same mutation has happened in the target gene. The likelihood of miRNA sequence similarity due to convergent evolution is considered very low, making them synapomorphic characters suitable for phylogenetic analyses (Thomson et al. 2014). Accordingly, many studies have used miRNA sequencing for resolving difficult phylogenetic questions, including a study supporting the monophyly of Panarthropoda, consisting of Tardigrada, Onychophora and Arthropoda (Campbell et al. 2011). Yet, some controversy over the usability of miRNAs for phylogenetic studies exists as sampling biases may have exaggerated the belief that miRNAs are rarely lost (Thomson et al. 2014), and as more whole genome sequences become available, it can be anticipated that the usefulness of miRNAs for phylogenetic inferences may decrease.

In addition to their use as phylogenetic characters, miRNAs have relevance for understanding gene regulation mechanisms governing developmental and physiological processes and they therefore represent a highly relevant and promising, yet relatively unexplored, avenue for research into tardigrade resilience. The presence or absence of specific miRNAs may be correlated with phenotypic traits, which can be used for identifying genes and proteins involved in specific processes. Notably, the cellular machinery used by miRNAs to regulate gene expression can be exploited to make gene knockdowns through the introduction, into a living organism, of double‐stranded RNA (dsRNA) molecules matching a given target gene sequence, which the cells then convert into miRNA‐like molecules called interfering RNAs (iRNAs) (Wilson and Doudna 2013). This principle can be used as a powerful tool to reveal gene functions by studying the phenotypic changes occurring after introducing the dsRNA. In tardigrades, this has been accomplished by microinjection of dsRNA to downregulate developmental genes (Tenlen et al. 2013), intrinsically disordered proteins (Boothby et al. 2017) and genes involved in stress response, including antioxidant enzymes and aquaporin water channels (Giovannini et al. 2022). Interestingly, Li et al. (2024) recently applied a soaking method to introduce dsRNA into tardigrades with the purpose of silencing a tardigrade‐specific gene and a putative horizontally transferred gene.

Alongside RNAi‐mediated knockdown of single genes, the ncRNA based CRISPR‐Cas genome editing tool, originating from the adaptive immune system of archaea and bacteria (Barrangou et al. 2007; Jinek et al. 2012), has emerged as a promising tool for targeting and modifying specific sites within the genome of living organisms (Wang et al. 2015; Adli 2018). Specifically, the Cas9 endonuclease can be used, together with a ca. 100‐nucleotide target‐specific single guide RNA (sgRNA), to make highly specific double‐strand breaks in genes within somatic as well as germline cells. The technique subsequently makes use of the endogenous cell DNA repair machinery to generate modifications in the genome. These modifications include loss‐of‐function mutations with deletions or insertions generated as a result of error‐prone non‐homologous end joining, as well as mutations with a defined insertion of exogenous DNA generated by homology‐directed repair. If the manipulations are made in germ cells or in a zygote or parthenote (many tardigrades reproduce by parthenogenesis) prior to initial DNA replication, they may be introduced into the germ line and thus inherited by future generations. The CRISPR‐Cas genome editing tool appears to work in most organisms in which it has been tried. Yet, tardigrade genome editing is still in its early stages and our own attempts, as well as those of other labs, have revealed that injections into tardigrade eggs with fine injection needles render the eggs undividable (Møbjerg et al. unpublished data). Consequently, injections and cell delivery methods can presently be done only in adult tardigrades (Tenlen et al. 2013) and, until recently, methods of introducing heritable traits into tardigrade germ cells have not been feasible. Interestingly, it has recently been shown that injections of CRISPR genome‐editing components into mature parthenogenetic tardigrade females could be a promising new method for heritable gene editing in tardigrades (Kumagai et al. 2022; Kondo et al. 2024).

## 2 Tardigrade Genome and Transcriptome Resources

Extant tardigrades comprise two major evolutionary lineages represented by the clades Eutardigrada and Heterotardigrada (Giribet and Edgecombe 2017; Jørgensen et al. 2018). Both clades contain highly tolerant species that readily enter cryptobiosis, but also less tolerant species. Whereas eutardigrades mainly thrive in permanent and ephemeral limnic microhabitats, heterotardigrades dwell in marine benthic, tidal and limno‐terrestrial microhabitats. Marine heterotardigrades are generally thought to be far less tolerant than species living in more extreme and changing habitats, such as tidal zones, mosses and lichens. Yet, little is currently known about stress tolerance among the highly diverse groups of marine heterotardigrades (Jørgensen and Møbjerg 2015), a field that deserves more attention.

To date, few genomes and transcriptomes of select tardigrade species have been sequenced, with most sequencing projects investigating eutardigrades only (Table 1). Hence, tardigrade sequencing is still in its infancy, with merely fifteen out of the currently ca. 1500 described species represented with publicly available genome or transcriptome sequences. Notably, sequences are lacking from 24 out of 31 presently recognised tardigrade families, including more ancestral marine heterotardigrade clades.

**TABLE 1:** Overview of tardigrade genome and transcriptome assemblies.

Phylogeny and taxonomy	Species	Habitat	Assembly	Publications
Eutardigrada, Parachela				
Hypsibioidea				
Hypsibiidae	*Hypsibius exemplaris*	Freshwater	Genome/transcriptome	Koutsovoulos et al. (2016), Yoshida et al. (2017)
	*Hypsibius henanensis*	Limno‐terrestrial	Genome/transcriptome	Li et al. (2024)
Acutuncidae	*Acutuncus antarcticus*	Limno‐terrestrial	Transcriptome	Giovannini et al. (2023), Anoud et al. (2024)
Ramazzottiidae	*Ramazzottius* cf. *varieornatus*	Limno‐terrestrial	Genome/transcriptome	Yoshida et al. (2017), Hashimoto et al. (2016)
	*Ramazzottius varieornatus*	Limno‐terrestrial	Transcriptome	Møbjerg et al. (2022), Neves et al. (2022a)
Macrobiotoidea				
Richtersiusidae	*Richtersius coronifer*	Limno‐terrestrial	Transcriptome	Stec et al. (2020)
	*Richtersius ingemari*	Limno‐terrestrial	Transcriptome	Kamilari et al. (2019)
Macrobiotidae	*Mesobiotus philippinicus*	Limno‐terrestrial	Transcriptome	Mapalo et al. (2020)
	*Paramacrobiotus fairbanski*	Limno‐terrestrial	Transcriptome	Anoud et al. (2024)
	*Paramacrobiotus metropolitanus*	Limno‐terrestrial	Genome	Hara et al. (2021)
	*Paramacrobiotus* cf. *richtersi*	Limno‐terrestrial	Transcriptome	Hara et al. (2021)
	*Paramacrobiotus spatialis*	Limno‐terrestrial	Transcriptome	Boothby et al. (2017)
Eutardigrada, Apochela				
Milnesiidae	*Milnesium inceptum*	Limno‐terrestrial	Transcriptome	Schokraie et al. (2010), Wang et al. (2014)
Heterotardigrada				
Echiniscoidea				
Echiniscoididae	*Echiniscoides sigismundi*	Marine tidal	Transcriptome	Kamilari et al. (2019)
Echiniscidae	*Echiniscus testudo*	Limno‐terrestrial	Genome/transcriptome	Borner et al. (2014), Mapalo et al. (2020), Murai et al. (2021), this study (GenBank, Bioproject: PRJNA1461411); see ‘Methods’ section for more detail

The first tardigrade genome to be sequenced was that of the eutardigrade *Hypsibius exemplaris*, previously known as *Hypsibius dujardini* (Boothby et al. 2015; Koutsovoulos et al. 2016; Yoshida et al. 2017; Gąsiorek et al. 2018). The species has a genome of ca. 104 Mb (Yoshida et al. 2017). *H. exemplaris* is a freshwater species that is easy to keep in laboratory culture—it reproduces via parthenogenesis and has a generation time of around two weeks when kept at room temperature and fed *Chlorococcum* or *Chlorella* spp. algae (Gabriel et al. 2007; McNuff 2018). The species is translucent, which makes it ideal for developmental studies, including studies involving various injection methods (Tenlen et al. 2013; Kumagai et al. 2022). These traits make *H. exemplaris* a clear candidate for becoming a new model organism (Goldstein 2018). Importantly, *H. exemplaris* is less tolerant of environmental stress compared to more xerophilous tardigrades that thrive in desiccation‐prone limno‐terrestrial habitats. For example, *H. exemplaris* does not tolerate acute desiccation, and it needs a slow desiccation protocol to successfully enter the tun state (Kondo et al. 2015; Poprawa et al. 2022). Recently, Li et al. (2024) sequenced another hypsibid tardigrade, *Hypsibius henanensis*, providing a well‐annotated chromosome‐level genome of 112.6 Mb. Like *H. exemplaris*, *H. henanensis* is moderately stress‐tolerant and reproduces via parthenogenesis.

In comparison, a highly stress‐tolerant limno‐terrestrial eutardigrade species, belonging to the *Ramazzottius varieornatus* species complex, has a compact genome of ca. 55 Mb (Hashimoto et al. 2016). Based on sequencing data, this Japanese *Ramazzottius* species is distinct from the European *R. varieornatus* (Figure 1), which was originally described by Bertolani and Kinchin (1993), and we therefore refer to it as *Ramazzottius* cf. *varieornatus* (Emdee et al. 2024). A recent phylogenetic analysis highlighted the presence of two previously unrecognised species complexes within the family Ramazzottiidae, yet this study did not focus on the *Ramazzottius varieornatus* species complex (Dey et al. 2024). Hence, much more work is still needed, especially as outdated species descriptions and missing genetic data still hinder reliable classification and evolutionary inference within this family (Dey et al. 2024). Notably, *Ramazzottius* species are among the most stress‐tolerant tardigrades, readily entering the quiescent tun state and enduring a range of extreme environmental conditions including cooling to milli‐Kelvin temperatures (Lee et al. 2022). Consequently, species in this genus have been a major subject of recent research on tardigrade resilience (Yamaguchi et al. 2012; Horikawa et al. 2013; Tanaka et al. 2015; Hashimoto et al. 2016; Heidemann et al. 2016; Hygum et al. 2017; Yoshida et al. 2017; Neves, Hvidepil, et al. 2020; Neves, Stuart, et al. 2020; Lee et al. 2022; Neves et al. 2022a, 2022b; Møbjerg et al. 2022; Al‐Ansari et al. 2024; Kondo et al. 2024). Like the two hypsibids mentioned above (*H. exemplaris* and *H. henanensis*), *R. varieornatus* (and the family Ramazzottiidae) belongs to the eutardigrade clade Hypsibioidea (Table 1) and like most limno‐terrestrial tardigrades, *R. varieornatus* reproduces by parthenogenesis, although males have occasionally been encountered (Møbjerg et al. unpublished data).

The relatively compact genome of *R*. cf. *varieornatus* could, at least to some extent, explain the enhanced stress tolerance compared to the two hypsibids, as smaller genomes likely involve lower levels of DNA damage and thus repair burdens following stress exposure (Einset and Collins 2018; Arakawa 2022). Importantly, genome size varies substantially among tardigrade species, and the fourth eutardigrade to have its genome sequenced, *Paramacrobiotus metropolitanus*, has a substantially larger genome of ~170 Mb (Hara et al. 2021; Sugiura et al. 2022). *P. metropolitanus* is a desiccation‐tolerant, limno‐terrestrial and dioecious species, which belongs to the clade Macrobiotoidea (see Table 1). Any putative correlation between genome size and stress tolerance among macrobiotoids remains to be investigated. Taken together, available data suggest that genome size could contribute to enhanced lineage‐specific stress tolerance alongside genome architecture (e.g., repeat content, gene family turnover, regulatory features) and stress‐responsive pathways as discussed in more detail below. In addition to the genomes of the four eutardigrades mentioned above, a genome of the stress tolerant limno‐terrestrial echiniscoidean heterotardigrade *Echiniscus testudo* was recently sequenced with an estimated genome assembly size of 110 Mb (Murai et al. 2021).

De novo transcriptome assemblies have been generated for an additional nine eutardigrades and two heterotardigrades (Table 1). Specifically, reference transcriptomes are available for the eutardigrades, *Acutuncus antarcticus* (Giovannini et al. 2023; Anoud et al. 2024), the European *Ramazzottius varieornatus* (Møbjerg et al. 2022), two species within the genus *Richtersius* (Kamilari et al. 2019; Stec et al. 2020), *Mesobiotus philippinicus* (Mapalo et al. 2020), three *Paramacrobiotus* species (Boothby et al. 2017; Hara et al. 2021; Anoud et al. 2024) and the apochelan *Milnesium inceptum* (Wang et al. 2014). Moreover, reference transcriptomes are available for the highly tolerant marine tidal echiniscoidean heterotardigrade *Echiniscoides sigismundi* (Kamilari et al. 2019) as well as for *Echiniscus testudo* (Mapalo et al. 2020; present study).

The assembled genomes and transcriptomes provide the basis for valuable insights into tardigrade survival strategies. They are also essential for contemporary studies on the evolution and phylogeny of Ecdysozoa (Borner et al. 2014; Giribet and Edgecombe 2017; Howard et al. 2022). Comparative genome and transcriptome analyses across the currently sequenced tardigrade species have revealed notable differences in gene repertoires, indicating that unique molecular adaptations have evolved within the various eutardigrade and heterotardigrade lineages (Kamilari et al. 2019; Fleming et al. 2024). It follows that a more comprehensive understanding of tardigrade extreme stress tolerance, and the mechanisms underlying cryptobiosis, will require sequencing of more genomes and transcriptomes across evolutionary lineages to find possible common genes and expression patterns among highly stress‐tolerant species. Interestingly, none of the many marine heterotardigrades, which may lack the ability to enter cryptobiosis, have yet been sequenced—hence, future sequencing projects should involve cryptobiotic as well as non‐cryptobiotic tardigrade species to elucidate possible differences in gene repertoires and expression profiles between highly stress‐tolerant tardigrades and tardigrades that do not tolerate extreme conditions.

## 3 Comparative Sequence and Differential Gene Expression Analyses

**FIGURE 3:**
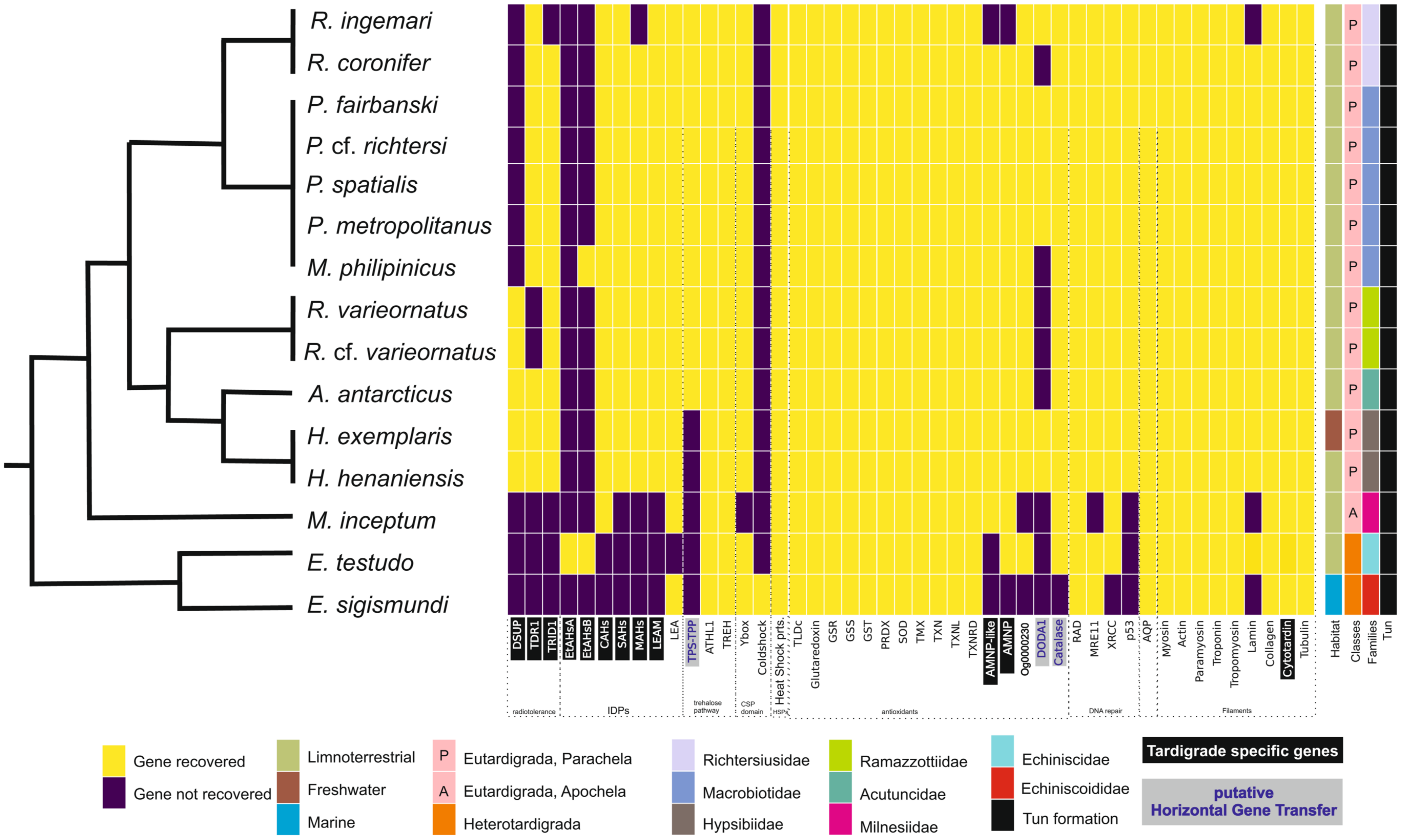
Distribution of stress tolerance‐related genes across tardigrade species. Phylogeny‐aligned presentation of the recovery or non‐recovery of candidate stress tolerance‐related genes across 15 tardigrade species (*cf*. Tables 1 and S2). Recovery was scored at the marker‐group level, with a gene family considered present when at least one predefined submarker/query sequence assigned to that group was recovered. Yellow = gene recovered; purple = not recovered. Right‐hand annotations indicate habitat (limno‐terrestrial, freshwater, marine), class or order (Parachela = P, Apochela = A, Heterotardigrada), family (colour‐keyed) and tun‐forming ability (black). The left tree provides display topology only (not time‐calibrated). Not recovered calls were made using tBLASTx, *E*‐value < 1*e*−04. See ‘Methods’ section and Workbooks S1 and S3 for more details. For gene abbreviations, see the abbreviation list.

Investigations of gene repertoires and stress‐induced expression changes provide important insights into the molecular basis of tardigrade stress tolerance. Accordingly, earlier EST resources, along with more recent mRNA‐seq studies, have enabled a systematic identification of candidate pathways and gene families responsive to stressors such as desiccation, osmotic stress, irradiation and temperature perturbation. These candidates include modules implicated in antioxidant defence, DNA protection and repair, proteostasis, transmembrane water transport and broader metabolic regulation (Møbjerg and Neves 2021; Arakawa 2022; Krakowiak et al. 2024; Li et al. 2024; Rolland et al. 2024). To synthesise these findings in a comparable framework, Figure 3 summarises recovery of a newly curated set of stress‐tolerance markers across the 15 tardigrade species for which genome and/or transcriptome assemblies are currently available (Tables 1, S1 and S2; Workbook S1). See ‘Methods’ section for more information on how the data mining and meta‐analysis were performed. Markers were selected to represent both conserved stress‐response modules and tardigrade‐specific candidate families, and to include structural stabilisation components given their plausible relevance to tun formation and maintenance (see discussion below). For multi‐copy gene families (e.g., heat shock proteins, cold‐shock domain factors, aquaporins), Figure 3 reports family‐level recovery (≥ 1 predefined submarker recovered, see Workbook S3), rather than copy number. We use the term ‘not recovered’ to indicate no detectable hit, under the criteria stated in the methods section and note that this should not be interpreted as a definitive absence of the specific marker from the species in question.

The first comprehensive investigations into tardigrade gene expression were based on EST data generated from the highly stress‐tolerant, carnivorous and parthenogenetic apochelan eutardigrade *Milnesium inceptum* (Schill et al. 2004; Förster et al. 2009, 2012; Mali et al. 2010; Reuner et al. 2010; Schokraie et al. 2010; Wang et al. 2014), previously known as *Milnesium tardigradum* (Morek et al. 2019). These investigations provided an initial overview of tardigrade gene repertoires focusing on transcripts involved in antioxidant defence, transmembrane water transport and molecular chaperoning (Schill et al. 2004; Förster et al. 2009, 2012; Mali et al. 2010; Reuner et al. 2010). While generating the first tardigrade reference transcriptome from *M. inceptum* and subsequently using this for mapping and quantifying expressed transcripts, Wang and co‐workers (2014) showed a modest change in transcription during entrance into, and exit out of, the desiccated tun state. Their analyses indicated down‐regulation of transcripts coding for proteins related to DNA replication, translation and protein degradation, supporting the notion of metabolic shutdown during entrance into anhydrobiosis. In addition, the authors reported up‐regulation, upon rehydration, of transcripts coding for selected chaperones and DNA repair proteins (Wang et al. 2014).

**FIGURE 4:**
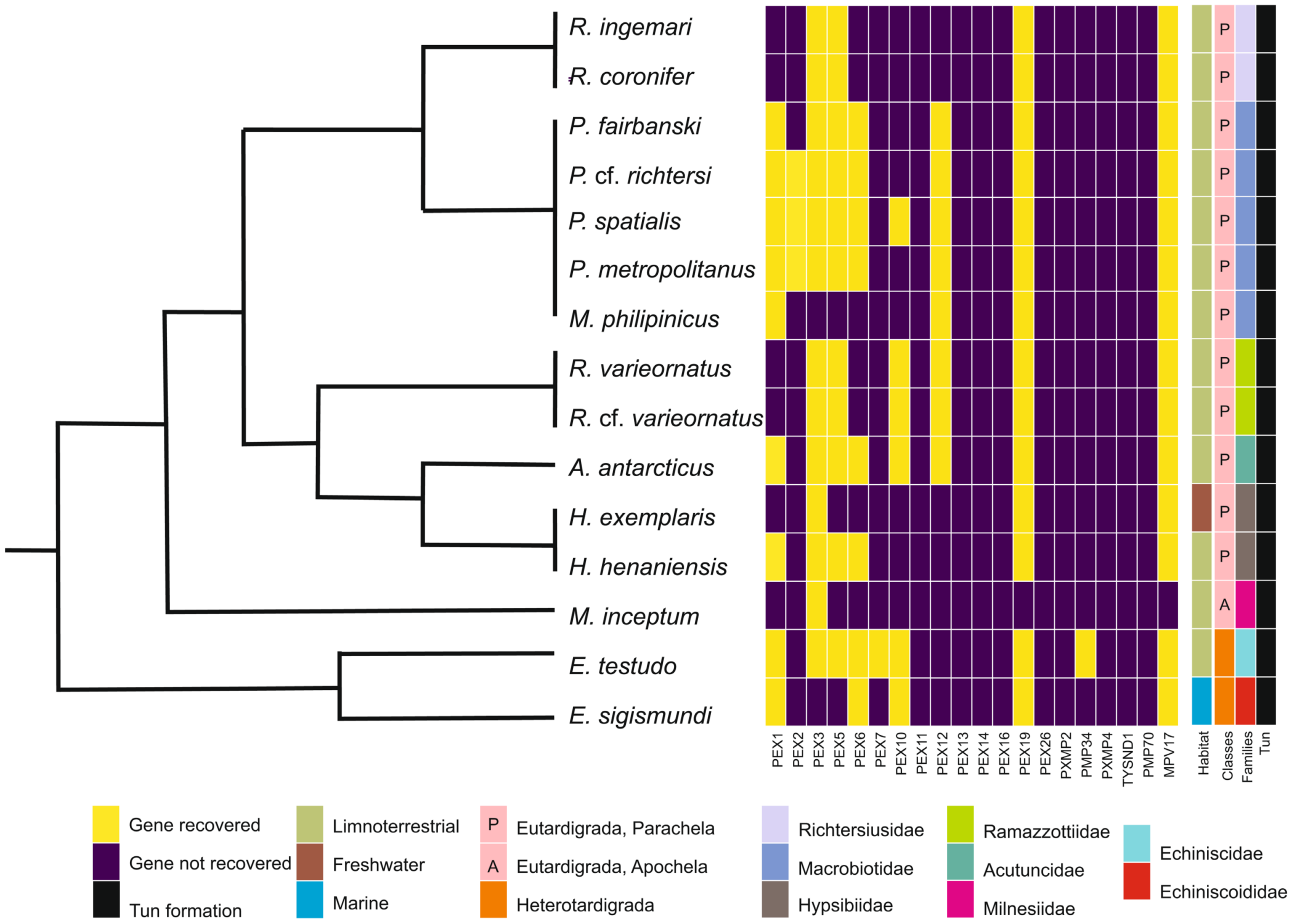
Distribution of peroxisome‐related genes across tardigrade species. Recovery or non‐recovery of peroxin genes across 15 tardigrade species (*cf*. Tables 1 and S2). Left: Species phylogeny (pruned from published trees; topology used for display only). Right: Heatmap showing genes (columns) scored as recovered (yellow) or not recovered (purple) per species (rows). Habitat, higher clade and family are indicated by side bars; tun‐forming ability is shown at the far right. Presence calls were made using tBLASTn, *E*‐value < 1*e*−04. See ‘Methods’ section and Workbooks S2 and S3 for more details. For gene abbreviations, see the abbreviation list.

Investigations based on more recently generated genome and transcriptome data have confirmed that tardigrades have a genetic toolkit consistent with universal metazoan gene contents (Hashimoto et al. 2016; Yoshida et al. 2017; Kamilari et al. 2019). Yet, tardigrades also seem to be among the animals that show the highest proportion of gene losses and gene gains (Guijarro‐Clarke et al. 2020), involving gene repertoires and expression profiles that may significantly differ from their sister groups (e.g., Mapalo et al. 2020; Chavarria et al. 2021). Intriguingly, the most stress‐tolerant tardigrades, such as various *Ramazzottius* species and the marine tidal heterotardigrade *Echiniscoides sigismundi* , express fewer genes within several stress‐related gene categories and all tardigrades seem to have lost genes within well‐known stress tolerance‐related pathways such as peroxisome biogenesis (Figure 4; Workbook S2; Hashimoto et al. 2016; Yoshida et al. 2017; Kamilari et al. 2019). All tardigrades, on the other hand, seem to have a comprehensive number of genes involved in antioxidant defence. Consistent with previous studies (Hashimoto et al. 2016; Yoshida et al. 2017; Kamilari et al. 2019), our current analyses reveal that most species possess the full complement of surveyed antioxidant defence related markers, underscoring the conservation and apparent physiological importance of this functional category (Figure 3; Figure S1). Notable exceptions are the expression of tardigrade‐specific Mn‐peroxidases (Yoshida et al. 2022: *AMNP* in Figure 3; Anoud et al. 2024: *AMNP‐like* in Figure 3), which we did not recover from selected tardigrades.

Whereas moderately stress‐tolerant species such as *Hypsibius exemplaris* and *Hypsibius henanensis* have a strong transcriptomic response to desiccation and radiation (Boothby et al. 2017; Yoshida et al. 2017; Anoud et al. 2024; Clark‐Hachtel et al. 2024; Li et al. 2024), highly stress‐tolerant tardigrades such as *Milnesium inceptum* and various *Ramazzottius* species constitutively express or only moderately change gene expression upon exposure to various stressors, including desiccation, radiation, high osmotic pressure and low temperatures (Wang et al. 2014; Hashimoto et al. 2016; Yoshida et al. 2017, 2022; Møbjerg et al. 2022; Emdee et al. 2024). Consequently, RNA‐seq datasets examining effects of environmental stress in highly stress‐tolerant tardigrades can easily be prone to batch effects, as non‐stress associated variables may be the main drivers for differential gene expression in these species (e.g., Emdee et al. 2024). Hence, an experimental protocol with a focus on eliminating non‐essential variables is paramount for detecting subtle expression changes in such species. Thus, both gene repertoire and transcriptomic responses depend on the tardigrade species. Accordingly, when embarking on tardigrade resilience and omics analyses, the choice of species as well as environmental stressor matters.

## 4 Molecular Mechanisms Underlying Tardigrade Resilience

Contemporary tardigrade sequence and differential expression analyses, together with investigations employing iRNAs, have led to three main theories explaining the origin of tardigrade resilience (Møbjerg 2025). This includes acquisition of genes through horizontal gene transfer (HGT), the evolution of genes specific for distinct tardigrade clades, as well as fine‐tuned expression of genes conserved among animals including tardigrades (Li et al. 2024; Møbjerg 2025). It is often assumed that the cryptobiotic abilities of tardigrades have been acquired twice during evolution due to selective pressures related to invasion of the terrestrial environment, that is, once in each of the evolutionary lineages leading to extant eutardigrades and echiniscid heterotardigrades (e.g., Kinchin 1994; Arakawa 2022; Yoshida and Tanaka 2022; Fleming et al. 2024). This assumption cannot explain why the phenomenon exists among marine heterotardigrade species such as *Echiniscoides sigismundi* , *Styraconyx haploceros* and *Archechiniscus* sp., which do not belong to either of these clades (Jørgensen and Møbjerg 2015; Hansen and Fujimoto 2018; Sørensen‐Hygum et al. 2018). Hence, a simpler explanation for the widely spread occurrence of cryptobiotic abilities among tardigrades is that tun formation and cryptobiotic survival is a basic tardigrade characteristic which has been retained in many extant taxa (Jørgensen and Møbjerg 2015; Hygum et al. 2016). It follows that the mechanisms underlying cryptobiosis in tardigrades likely evolved only once, but that these mechanisms subsequently may have been refined within the various evolutionary lineages. Consequently, tun formation and cryptobiosis likely evolved in a marine tardigrade ancestor and osmobiosis, induced by a sudden rise in external osmotic pressure likely represents the evolutionary forerunner of other cryptobiosis sub‐states (Hvidepil and Møbjerg 2023; Emdee et al. 2024). As discussed in more detail below, it is worth noting that the currently recognised tardigrade‐specific genes (e.g., Hashimoto et al. 2016; Boothby et al. 2017) seem to be restricted to certain species and clades (Figure 3; Kamilari et al. 2019; Murai et al. 2021). This also holds for genes that potentially were acquired through HGT (e.g., Hara et al. 2021; Li et al. 2024). Hence, these genes may enhance tolerance in specific species and clades, but they cannot explain the general resilience found across tardigrade evolutionary lineages (Møbjerg 2025).

As outlined in more detail below, the genes proposed to be involved in tardigrade stress tolerance support multiple mechanisms that likely act synergistically, thereby providing the basis for protection across biological organisation levels. Notably, adaptations that protect against desiccation likely result in cross‐tolerance and may mitigate damage from other stressors such as irradiation and thermal stress (e.g., Jönsson 2019; Yoshida et al. 2022; Loeffelholz et al. 2024). This is mechanistically plausible because multiple damage modes overlap across stresses, including oxidative stress, DNA lesions, protein unfolding and aggregation and membrane destabilisation. Accordingly, the protective modules discussed below, for example, ROS management, DNA protection and repair, molecular chaperones including various intrinsically disordered proteins (IDPs) and cellular structural stabilisation, are likely reused across stress contexts, although the strength of experimental support varies among species and stress paradigms. Explicit cross‐stress experimental designs will in the future be important to disentangle shared versus stressor‐specific mechanisms.

### 4.1 Tardigrade‐Specific Genes

Comparative sequence analyses indicate that tardigrade proteins may have an abundance of intrinsic disordered regions with potential importance for extreme stress tolerance (Boothby et al. 2017; Kamilari et al. 2019; Arakawa 2022). In 2012, Yamaguchi and co‐workers reported the presence in *Ramazzottius* cf. *varieornatus* of two abundantly expressed heat‐soluble protein families with predicted large stretches of disordered regions CAHS (Cytoplasmic Abundant Heat Soluble) and SAHS (Secretory Abundant Heat Soluble). Subsequently, Tanaka and co‐workers (2015) described MAHS (Mitochondria Abundant Heat Soluble) and RvLEAM ( *R. varieornatus* Late Embryogenesis Abundant Mitochondrial) from this species. The release of a genome sequence for *R*. cf. *varieornatus*, along with the report of high expression of genes including *CAHS* and *Dsup* (Damage suppressor) (Hashimoto et al. 2016), resulted in great attention and research into the possible function of tardigrade‐specific genes (reviewed in Arakawa 2022). Yet, Kamilari et al. (2019) were unable to find transcripts from any of these genes, that is, *CAHS*, *SAHS*, *MAHS*, *RvLEAM* and *Dsup*, in a reference transcriptome from the heterotardigrade *Echiniscoides sigismundi* . The observation that heterotardigrades may be missing these genes altogether was later supported by sequencing of a transcriptome and a genome of the heterotardigrade *Echiniscus testudo* (Mapalo et al. 2020; Murai et al. 2021). With this review we also provide a new *E. testudo* transcriptome (see ‘Method’ section and Table S1), and using our current search pipeline we confirm that homologues of *CAHS*, *SAHS*, *MAHS*, *RvLEAM* and *Dsup* indeed seem to be absent across heterotardigrade assemblies (Figure 3; Workbook S1). Furthermore, our analyses support earlier comparative transcriptome and genome analyses indicating that *Dsup*, a gene presumed to be involved in radiotolerance, is restricted to selected hypsibioidean eutardigrades, and that *SAHS, MAHS* and *RvLEAM* are limited to the eutardigrade clade Parachela, whereas *CAHS* genes are present among all eutardigrades (Table 1; Figure 3; Kamilari et al. 2019; Anoud et al. 2024; Fleming et al. 2024). We emphasise that non‐recovery of specific genes and transcripts should not be interpreted as a definitive absence, especially when it comes to fast‐evolving genes such as *Dsup*. Interestingly, Sugiura et al. (2024) recently reported the presence of a *Dsup* ortholog in the genome of *Paramacrobiotus metropolitanus* based on synteny with the *Hypsibius exemplaris Dsup* locus, despite a lack of detectable BLAST similarity at standard thresholds. Accordingly, we note that our BLAST‐based recovery matrix should be interpreted as conservative for *Dsup* and similar IDPs.

Murai and co‐workers (2021) recently suggested that heterotardigrades and eutardigrades independently acquired heat‐soluble proteins, with significance for cryptobiotic survival, through convergent evolution, a hypothesis that requires further examination. Specifically, the authors identified two families of abundant heat‐soluble proteins (EtAHS) in the genome of the limno‐terrestrial heterotardigrade *Echiniscus testudo* . The authors propose that these proteins are important for desiccation tolerance throughout Heterotardigrada (Murai et al. 2021; Arakawa 2022), and they report that *EtAHS* transcripts are present in the transcriptome of *Echiniscoides sigismundi* . In contrast to the results of Murai et al. (2021), we do not find any evidence of *EtAHS* sequences in the transcriptome assembly of *E. sigismundi* . Accordingly, the hypothesis that these proteins are important for desiccation tolerance throughout Heterotardigrada is not supported by available sequencing data. Surprisingly, we found an *EtAHS* sequence in the transcriptome of the eutardigrade *Mesobiotus philippinicus* (Figure 3). This sequence is likely a result of contamination, as it has a high similarity to an *EtAHS* sequence from GenBank identified as *Viridiscus perviridis*, a species which is relatively closely related to *Echiniscus testudo* and thus belongs to the clade Echiniscidae. In order to substantiate the results of Murai et al. (2021), here we provide a new transcriptome for *Echiniscus testudo* (see ‘Methods’ section and Table S1) in which we confirm the presence of *EtAHS*. In summary, our current analyses indicate that *EtAHS* seem to be restricted to the heterotardigrade family Echiniscidae, that is, they are not a general feature of heterotardigrades and their putative role in stress tolerance and cryptobiotic survival remains to be elucidated.

### 4.2 Formation and Stabilisation of the Tun State

Formation of the tun state (Figure 1) is a powerful adaptation to extreme and rapidly changing environments, ultimately preparing the organism for decades in cryptobiosis. Tun formation relies on an active, energy‐requiring, muscle‐driven compaction of the body involving structural reorganisation across biological scales. Longitudinal body and leg musculature contract to shorten the body axis and withdraw appendages, while visceral muscles facilitate the tugging in of organs (Halberg et al. 2013; Møbjerg et al. 2018). Anhydrobiotic tun formation is associated with a significant reduction in body volume, and during desiccation tardigrades may lose almost all body water (Crowe 1972). While cells undergo shrinkage with water leaving the cytoplasm through aquaporin water channels (Figure 3: AQP; Grohme et al. 2013), the cytoplasm becomes increasingly electron dense, yet organelle and overall cellular structure remain broadly recognisable in species, such as *Richtersius ingemari* and *Ramazzottius* cf. *varieornatus* (Czerneková et al. 2017; Galas et al. 2024). In contrast, ultrastructural investigations of desiccated *Hypsibius exemplaris* tuns revealed aberrant cellular and extracellular structures (Richaud et al. 2020; Poprawa et al. 2022), with the severity of cellular damage depending on dehydration protocol and duration of the anhydrobiotic state (Poprawa et al. 2022). Accordingly, here we iterate the conclusion by Poprawa et al. (2022) that *H. exemplaris* is not a good model for research into desiccation tolerance and the mechanisms underlying anhydrobiosis. Yet, in more desiccation tolerant tardigrade species, scaffolds across biological scales, that is, from the whole animal to subcellular levels, seem to fortify the tun enabling it to withstand dehydration‐induced deformation and ensure that structural integrity is preserved throughout cryptobiosis. If three‐dimensional structure cannot be preserved, the animal loses its ability to return to life.

Scaffolds supporting three‐dimensional tun‐structure seem to rely on the expression of evolutionary conserved, but also tardigrade‐specific, genes. Together with contributions from stress protectants, such as small heat shock proteins (HSPs) and CAHS, the cuticle and filament‐forming proteins of the extracellular matrix and cytoskeleton stabilise tun‐architecture and enable reversible recovery upon removal of the environmental stressor. We propose that supporting scaffolds with significance for upholding 3D tun structure include (i) the chitin‐containing cuticle (Baccetti and Rosati 1971; Greven and Greven 1987; Kristensen and Neuhaus 1999; Czerneková and Vinopal 2021), which contributes to mechanical support at whole organism and organ levels, (ii) collagen‐containing extracellular matrices that support organs and tissues (Baccetti and Rosati 1971; Exposito et al. 2002; Massa et al. 2024) and (iii) the cytoskeleton [including microtubules, microfilaments (F‐actin), and intermediate‐filament‐like elements] that supports cellular and nuclear compartments.

#### 4.2.1 Filament‐Forming Proteins

Filament‐forming proteins of the tardigrade cytoskeleton include microfilaments and conserved contractile systems (actin–myosin with connectin and regulatory cofactors), microtubular networks and intermediate‐filament‐like elements. Actin, the most abundant protein within eukaryotic cells (Pollard 1990), supplies tardigrade cells with a prominent intracellular scaffold which, along with myosin, generates the contractile force necessary for tun entry. Specifically, myosin motors interact with actin in tardigrade muscle cells to drive the longitudinal body contraction and leg retraction that produce the tun. Actin and myosin filaments further provide a basis for stabilising the three‐dimensional structure of the tun, with filaments likely locking in a rigour state following metabolic shutdown, ensuring preservation of three‐dimensional structure during cryptobiosis (Møbjerg and Neves 2021). The noticeable appearance of actin, especially within tardigrade muscle cells, has been visualised in numerous investigations using F‐actin phalloidin stainings (Schmidt‐Rhaesa and Kulessa 2007; Schulze and Schmidt‐Rhaesa 2011; Halberg et al. 2009, 2013; Marchioro et al. 2013; Smith and Jockusch 2014; Gross and Mayer 2019; Persson et al. 2019). Also, proteomic surveys of *Milnesium inceptum* identified abundant actin in both active and tun states, consistent with a structural role through dehydration and recovery (Schokraie et al. 2012). Noticeably, in a study based on cDNA libraries from the eutardigrade *Hypsibius klebelsbergi* , Prasath et al. (2012) found no indication of special modifications of tardigrade calcium‐binding proteins or regulation of actin‐myosin interactions. Accordingly, the authors concluded that the well‐known, conserved dual‐regulation consisting of myosin light‐chain phosphorylation and calcium‐dependent access of myosin to actin, which is controlled by a tropomyosin‐troponin complex, also applies to tardigrade somatic muscles. Examining the available assemblies (Tables 1 and S2), we confirm the presence of actin, myosin, troponin and tropomyosin throughout the sequenced tardigrade species (Figure 3). Moreover, paramyosin, a hallmark invertebrate thick‐filament protein that stabilises the myosin core and increases filament stiffness (Yang, Zhao, et al. 2022) is also present throughout the tardigrades (Figure 3; Schokraie et al. 2012).

In addition to actin‐ and myosin filament‐based scaffolds, microtubule networks (Floriančičová et al. 2023) likely provide compressive support and tracks for targeted trafficking during tun entry and exit. Microtubules may further organise and preserve organelle positioning and, when stabilised, help maintain cellular architecture. Specifically, stabilisation (e.g., increased acetylation, detyrosination and reduced turnover) can limit network collapse as water is lost from the cells, while crosstalk with actin (via shared regulators and cross‐linkers) may coordinate contraction and intracellular architecture. The presence of a prominent microtubule network throughout tardigrades has been visualised in numerous investigations of tardigrade nervous systems using primary antibodies directed against especially acetylated α‐tubulin (Zantke et al. 2008; Persson et al. 2012, 2014; Mayer, Kauschke, et al. 2013; Mayer, Martin, et al. 2013; Schulze and Schmidt‐Rhaesa 2013; Schulze et al. 2014; Smith et al. 2018). Accordingly, in the present study we retrieved tubulin sequences from all of the 15 tardigrade species with available assemblies (Figure 3).

Intermediate filament‐like elements include a lamin‐based nuclear lamina, and also the tardigrade‐specific lamin‐derived, cytoplasmic protein cytotardin, which forms belt‐like cortical filaments in epithelial cells (Hering et al. 2016). Cytotardin likely offers mechanical strengthening of epithelia during desiccation and participates in maintaining tun integrity (Hering et al. 2016; Tanaka et al. 2022). Notably, cytotardin is encoded in the genomes and expressed in the transcriptomes of all investigated tardigrades (Figure 3).

Besides cytotardin and the well‐known cytoskeleton proteins mentioned above, Al‐Ansari et al. (2024) recently investigated the induction of sHSPs in *R. varieornatus* and found that the major inducible sHSP, HSP20‐3, is an active chaperone that forms filament‐like structures which potentially could protect tardigrade cells during heat stress and support phase separation during desiccation. Also, it has recently been proposed that the eutardigrade‐specific CAHS proteins are in fact cytoskeleton‐like proteins that form filamentous networks during desiccation, thereby contributing to the stabilisation of cell integrity during tun formation (Tanaka et al. 2022).

### 4.3 Defence Against Free Radicals

A common strategy across stress‐tolerant tardigrade species seems to involve minimising potential damage to cells and tissues through scavenging of reactive oxygen species (ROS) and other free radicals that are generated during exposure to stressors such as ionising radiation (IR) and desiccation (Rizzo et al. 2010; Yoshida et al. 2022; Anoud et al. 2024; Clark‐Hachtel et al. 2024). It is apparent from the sequencing data reported so far that all tardigrades express a comprehensive number of genes involved in antioxidant defence with noticeable expansions within soluble glutathione S‐transferases, and particularly superoxide dismutases (Figure 3; Figure S1; Förster et al. 2012; Hashimoto et al. 2016; Hygum et al. 2017; Yoshida et al. 2017; Kamilari et al. 2019; Murai et al. 2021). In addition, mitigation of oxidative stress damage in many tardigrades may involve expression of tardigrade‐specific Mn‐dependent peroxidases (Figure 3; Yoshida et al. 2022; Anoud et al. 2024). Furthermore, most but not all tardigrades seem to express catalases (CAT, Figure 3; Kamilari et al. 2019) and sequence comparisons among parachelan eutardigrades indicate that tardigrades may have obtained their catalase genes through HGT (Hashimoto et al. 2016; Yoshida et al. 2017; Kamilari et al. 2019; Murai et al. 2021). Interestingly, irradiated *Hypsibius henanensis* upregulate a putative horizontally transferred gene (*DODA1*) that encodes an enzyme (a dihydroxyphenylalanine dioxygenase) involved in the biosynthesis of betalains, which are pigments with free radical scavenging properties (Li et al. 2024). Our current investigation of available assemblies indicates that *DODA1* is present among various clades of parachelan eutardigrades (Table 1; Figure 3). Hence, defence against the potentially damaging effects on biomolecules of free radicals is likely an important mechanism underlying tardigrade extreme stress tolerance. The genes involved seem to include genes coding for detoxifying enzymes, such as superoxide dismutases which are common to all tardigrades, as well as tardigrade‐specific and horizontally transferred genes that enhance defence in specific species and clades.

### 4.4 Peroxins and Peroxisome Biogenesis

The biogenesis and function of peroxisomes, which are multifunctional organelles involved in lipid oxidation and subsequent detoxification of ROS, depend on conserved peroxin (*PEX*) genes (Farré et al. 2019; Kumar et al. 2024). Proteins associated with peroxisomes contain targeting sequences, known as PTS1 and PTS2, which are recognised by cytosolic receptors PEX5 and PEX7, which in turn interact with peroxisomal import machineries.

As noted above, and in line with earlier studies (Hashimoto et al. 2016; Yoshida et al. 2017; Kamilari et al. 2019), we recovered a reduced and patchy peroxisomal gene repertoire across the 15 tardigrade species with available genome and/or transcriptome assemblies (Table 1; Figure 4; Figure S2). A core PEX3–PEX19 membrane biogenesis route (Fang et al. 2004; Farré et al. 2019; Skowyra and Rapoport 2022) seems to be broadly retained within tardigrades, with *PEX3* and *PEX19* found in 13 and 14 species, respectively, and *PEX5* (the PTS1 receptor) retrieved from 11 species (Figure 4, Figure S2). In contrast, the PTS2 pathway was not recovered from any tardigrade species, except *E. testudo* which seems to carry *PEX7* (PTS2 receptor gene). Specifically, the docking components *PEX13* and *PEX14* (Barros‐Barbosa et al. 2019; Wang et al. 2019), as well as the PEX1/PEX6 membrane anchor PEX26, were not recovered from any of the screened tardigrade genomes or transcriptomes (Figure 4; Figure S2). Elements of the PTS receptor recycling machinery (Law et al. 2017) were recovered sporadically, with *PEX1* and *PEX6* found in 9 and 8 species, respectively. The RING E3 ligase components show stronger lineage restriction (*PEX2* in 3/15 species; *PEX10* in 6/15; *PEX12* in 8/15 species). *PEX11* and *PEX16* were not recovered from any species (Figure 4; Figure S2). Peroxisomal transporters and processing factors were also largely unrecovered, that is, *PXMP2*, *PXMP4*, *PMP70* (*ABCD3*) and *TYSND1* (matrix protease gene) were not recovered across the dataset; however, *PMP34* was recovered from *E. testudo* , distinguishing this species from the others by retention of at least one peroxisomal metabolite transporter. Finally, MPV17 (a factor linked to redox/homeostasis crosstalk) was recovered from all species except *Milnesium inceptum* (Figure 4; Figure S2).

The pattern described above is consistent with other ecdysozoans, such as *Caenorhabditis elegans* , that have secondarily streamlined peroxisomal matrix import. Specifically, *C. elegans* lacks the PTS2 pathway and does not encode TYSND1, with many former PTS2 cargos retargeted via PTS1 (Motley et al. 2000). MPV17, involved in redox and homeostasis crosstalk (Spinazzola et al. 2006), was recovered from all the investigated tardigrade species other than *Milnesium inceptum* (Figure 4; Figure S2). Overall, the pattern is consistent with a minimal, PTS1‐based import system, with lineage‐specific retention and broad losses affecting docking, PTS2 import, division and several membrane transporters. Notably, *E. testudo* seems to carry the most complete gene set, including *PEX7*, which appears absent in the other tardigrades (Figure 4; Figure S2). The apparent gene absence across the investigated tardigrade species could reflect true secondary loss, as documented for the PTS2 pathway in *C. elegans* , and the reduced and ambiguous PTS2 usage in *Drosophila* (Faust et al. 2012), but it could potentially also reflect extreme sequence divergence and annotation gaps. Notably, *PEX13* and *PEX14* are fast‐evolving and can be missed by standard homology searches, and animal *PEX26* is lineage‐specific (Gardner et al. 2018). We therefore currently interpret these as not recovered rather than definitive absences, and recommend targeted HMM domain searches, synteny checks and read mapping around candidate loci.

Functionally, a curtailed peroxin gene repertoire suggests limited peroxisomal β‐oxidation and metabolite exchange, with ROS handling and lipid remodelling potentially routed through mitochondria and cytosolic pathways, consistent with known peroxisome‐mitochondrion crosstalk (Demarquoy and Le Borgne 2015) and the strong antioxidant gene complements in tardigrades (see above). This interpretation aligns with earlier comparative genomics and transcriptomics studies, indicating peroxisome‐pathway gene loss across tardigrade lineages (Hashimoto et al. 2016; Yoshida et al. 2017; Kamilari et al. 2019), yet our current dataset highlights a relatively complete module in *E. testudo* , underscoring a potential lineage‐specific retention within Tardigrada.

### 4.5 DNA Protection and Repair

Although tardigrades seemingly devote substantial effort to antioxidant defence, thereby minimising potential macromolecular damage, post‐stress recovery also clearly depends on repair pathways (Kamilari et al. 2026). Specifically, it is well known that tardigrades in their desiccated tun state are prone to DNA damage, and that considerable repair of DNA—and likely other macromolecules—occurs as they awake from the tun state (Neumann et al. 2009; Rebecchi et al. 2009). The latter also applies to active tardigrades recovering from severe radiation exposure (Horikawa et al. 2013; Jönsson 2019; Anoud et al. 2024; Clark‐Hachtel et al. 2024; Li et al. 2024). Hence, protection and repair of DNA are important aspects of tardigrade resilience. Recent investigations in selected eutardigrades have provided glimpses into the nature of such mechanisms, suggesting that DNA‐associating proteins such as Dsup shield DNA in hypsibioideans from radiation and hydroxyl radicals (Hashimoto et al. 2016), and TDR1 (Tardigrade DNA damage Response protein 1) confers resistance to ionising radiation in both hypsibioidean and macrobiotid tardigrades (Figure 3; Anoud et al. 2024). Also, Li et al. (2024) recently reported upregulation of a double‐strand break repair‐promoting protein, TRID1 (tardigrade‐specific radiation‐induced disordered protein 1), in *Hypsibius henanensis* following carbon ion radiation. Our current analyses indicate that *TRID1* seems to be lacking in heterotardigrades, but we retrieved sequences of the gene from all investigated parachelan eutardigrades except *Richtersius ingemari* (Figure 3; Workbook S1). Hence, the mentioned proteins (Dsup, TDR1 and TRID1) appear specific to selected parachelan eutardigrade clades.

Comparative sequence analyses have further shown that all tardigrades sequenced so far express well‐known genes within single‐strand repair mechanisms, such as the mismatch and nucleotide excision repair systems, with some components appearing expanded (Kamilari et al. 2019). In addition, all parachelan eutardigrades express a homologue of *p53* and a number of components within the base excision repair (BER) pathway, as well as most components within both major pathways of double‐strand repair, that is, homologous recombination (HR) and nonhomologous end‐joining (NHEJ) (Kamilari et al. 2019). It has been shown that gamma radiation in *M. inceptum* leads to increased expression of *Rad51 recombinase* involved in HR repair (Beltrán‐Pardo et al. 2013), and recent studies in *Hypsibius exemplaris* have revealed wide upregulation of transcripts from BER as well as HR and NHEJ pathways following exposure to gamma radiation (Anoud et al. 2024; Clark‐Hachtel et al. 2024). Also, Li et al. (2024) found upregulation of mitochondrial respiratory chain enzymes (BCS1 and NDUFB8) following irradiation of *Hypsibius henanensis*, and propose that these enzymes exert radioprotective effects through regeneration of NAD+, which is required for protein poly ADP‐ribosylation involved in DNA damage repair. Interestingly, *Echiniscoides sigismundi* seems to lack expression of several important transcripts within BER and the above‐mentioned double‐strand repair mechanisms, including *p53* and all essential components of NHEJ, indicating that this heterotardigrade may use alternative pathways for repairing double‐strand breaks (Kamilari et al. 2019). Notably, our current analyses show that the apparent lack of *p53* also applies to *Echiniscus testudo* (see Figure 3 and Workbook S1), indicating that loss of this vital regulator of genome stability could perhaps be a more general heterotardigrade feature. The latter observation clearly deserves further validation. In summary, a detailed understanding of the mechanisms underlying tardigrade DNA protection and repair remains elusive.

### 4.6 Trehalose Biosynthesis

Experiments on the molecular adaptations underlying tardigrade stress tolerance have indicated that eutardigrades belonging to Parachela can accumulate trehalose during desiccation (Westh and Ramløv 1991; Hengherr et al. 2008; Jönsson and Persson 2010). Notably, this non‐reducing disaccharide may accumulate to very high concentrations in, for example, desiccated brine shrimp embryos and sleeping chironomid larvae (Clegg and Jackson 1992; Cornette and Kikawada 2011). The sugar likely protects membranes and preserves macromolecule structure by replacing water and by vitrification, respectively (Crowe et al. 1998; Sakurai et al. 2008). However, neither the apochelan eutardigrade *Milnesium inceptum* nor heterotardigrades seem to accumulate trehalose (Hengherr et al. 2008), and Mali et al. (2010) reported that transcripts of trehalose‐6‐phosphate synthase genes (*TPS*), which are involved in trehalose biosynthesis, are absent from the *M. inceptum* ESTs. This would suggest that *M. inceptum* is incapable of synthesising trehalose. This observation was later supported by the sequencing of the first heterotardigrade transcriptome (Kamilari et al. 2019). Specifically, Kamilari et al. (2019) found no evidence in the *Echiniscoides sigismundi* transcriptome of *TPS* genes, which potentially could support the hypothesis that parachelan eutardigrades may have acquired genes involved in trehalose biosynthesis through HGT (Yoshida et al. 2017; Hara et al. 2021). We note that Nguyen et al. (2022) recently proposed that trehalose and CAHS proteins work synergistically to promote desiccation tolerance in *Hypsibius exemplaris*. However, we were unable to retrieve *TPS* and trehalose‐6‐phosphate phosphatase (*TPP*) genes from the genomes of *H. exemplaris* as well as *H. henanensis*, suggesting that hypsibiid tardigrades lack these genes (Figure 3). Moreover, in accordance with previous studies, our survey across tardigrade assemblies suggests that these genes may also be missing from *M. inceptum* and the heterotardigrades *E. sigismundi* and *Echiniscus testudo* . Taken together, trehalose accumulation does not seem to be vital for cryptobiotic survival in tardigrades, but could indeed enhance extreme stress tolerance in selected parachelan species.

### 4.7 Heat Shock Response and Heat Shock Proteins

Even the most stress‐tolerant tardigrades are extremely sensitive to high temperatures, with 50% mortality temperatures for active animals in the range of 35°C–40°C (e.g., Neves, Hvidepil, et al. 2020; Neves, Stuart, et al. 2020; Kayastha et al. 2024; Loeffelholz et al. 2024). Accordingly, heat‐stressed (35°C) *Ramazzottius varieornatus* show a major shift in transcription, with transcripts of several HSPs being upregulated, including two HSP70 isoforms, one HSP90, one HSP68, one HSP60 and four small HSPs (Neves et al. 2022a, 2022b). HSPs are ubiquitous and highly conserved proteins present in both prokaryotes and eukaryotes—they are essential for cellular protection against stress, ensuring proper protein folding and preventing cellular damage (Dubrez et al. 2020; Nesmelov et al. 2018; Richter et al. 2010). Large HSPs help refold misfolded proteins and assist in their proper folding, while sHSPs generally prevent protein aggregation by binding and stabilising misfolded proteins (Richter et al. 2010). As noted above, Al‐Ansari et al. (2024) recently investigated the induction of sHSPs in *R. varieornatus* and showed that the major inducible sHSP, HSP20‐3, is an active chaperone that forms filament‐like structures. Hibshman et al. (2023) found that overexpression of sHSPs from *Hypsibius exemplaris* in bacterial cells improved bacterial desiccation survival, leading the authors to argue for an involvement of sHSPs in tardigrade desiccation tolerance.

Schill and co‐workers (2004) provided evidence of a classical heat shock response in *Milnesium inceptum* involving expression of three HSP70 isoforms, and further showed that one of these isoforms was induced during entrance into anhydrobiosis. On the other hand, expression analyses by Reuner et al. (2010) suggested a limited role for HSPs in the desiccation tolerance of *M. inceptum*—an observation that was corroborated by expression analyses in *Paramacrobiotus richtersi* (Rebecchi et al. 2009; Rizzo et al. 2010). However, Kayastha et al. (2024) recently found that active state *Paramacrobiotus experimentalis*, upon an increase in external temperature, increases the levels of HSPs (HSP27, HSP60 and HSP70). Additionally, HSP70 levels increased in *Richtersius ingemari* after exposure to heat and ionising radiation and during rehydration from the desiccated tun state (Jönsson and Schill 2007) and an HSP70 transcript was upregulated in *Ramazzottius varieornatus* in response to rising external osmolyte concentrations (Emdee et al. 2024). Hence, expression analyses of HSPs, such as HSP70 and HSP90 and various sHSPs, suggest that these molecular chaperones contribute to molecular protection during heat shock and possibly cryptobiosis in tardigrades. Interestingly, as compared to other tardigrades, the moderately stress‐tolerant *Hypsibius exemplaris* has an exceptionally high number of *HSP70* genes (Yoshida et al. 2017), which may be related to its more limited stress tolerance (Kamilari et al. 2019). Our current investigation suggests that this also seems to be the case for *Hypsibius henanensis*.

### 4.8 Cold Shock Proteins

Cold shock proteins (CSPs), including the eukaryotic Y‐box (YB) proteins, are conserved nucleic acid–binding proteins that act as RNA chaperones and help stabilise and regulate transcripts under stress conditions such as temperature fluctuations. Kamilari et al. (2019) retrieved a highly expressed transcript of a novel bacterial‐style CSP from the heterotardigrade *Echiniscoides sigismundi* and further showed that YB proteins are present across tardigrade lineages. These proteins may be involved in RNA chaperoning and regulation of translation during cooling (Kamilari et al. 2019), and may therefore play an important role in cryobiotic survival by regulating gene products involved in preparing tardigrades for extracellular freezing (Møbjerg et al. 2022).

CSPs are united by a highly conserved Cold Shock Domain (CSD; ~70 aa with RNA recognition motifs) that binds to single‐stranded nucleic acids and confers RNA‐chaperone activity (Kleene 2018). In metazoans, CSD‐containing proteins are typically YB family members (and, in some lineages, Lin28) which also have low‐complexity, arginine‐ and glycine‐rich C‐terminal segments that broaden regulatory roles beyond acute cold shock (Heinemann and Roske 2021). Across the investigated tardigrade species, YB proteins were broadly recovered, but lineage‐specific deviations are notable (Figure 3). Specifically, as noted above, *Echiniscoides sigismundi* has an additional, small CSP sequence, comprising essentially only the CSD, coexisting with transcripts of the canonical YB protein (Kamilari et al. 2019). The small CSP sequence is consistent with the truncation of an YB ancestor and suggests a highly advanced, bacteria‐style RNA‐chaperone involved in rapid cold responses. In contrast, we did not detect canonical *YB* or *CSP* orthologs in *Milnesium inceptum* (Figure 3), potentially implying a reliance on alternative RNA‐binding factors (e.g., other RNA‐binding proteins, such as Lin28‐like proteins) or, alternatively, condition‐specific expression resulting in a lack of CSD transcripts from the available EST‐based data. Hence, given the strong conservation of the CSD among tardigrades, we interpret this apparent non‐recovery cautiously, as it may reflect life‐stage, tissue or stimulus‐dependent expression or incomplete sampling and assembly.

## 5 Future Directions

The acquisition of new high‐quality chromosome‐level reference genomes as well as transcriptomes will, in the future, provide the basis for a far better understanding of tardigrade evolution, diversity and adaptation. New genome and transcriptome data across evolutionary lineages will be crucial for phylogenetic analyses, biodiversity surveys, and, importantly, investigations into the mechanisms underlying tardigrade resilience. Specifically, when genomes and transcriptomes become available for many more species, it will become easier to map gene repertoires to phenotypic characters and hence make predictions about gene function. In addition, corroborating current approaches and datasets with other omics data, including proteomics and metabolomics, will add a further level of complexity involving post‐transcriptional regulation. Importantly, it is highly likely that tardigrade resilience involves epigenetic mechanisms, including heritable changes in gene expression that occur without altering the underlying DNA sequence (Dupont et al. 2009). These changes are often driven by modifications such as DNA methylation, histone modification and ncRNAs, which collectively influence how genes are turned on or off in response to environmental stimuli (Bartel 2004; Dupont et al. 2009). To date, there is a complete absence of studies investigating epigenetic mechanisms in tardigrades. Hence, the omics approach, involving genomics, transcriptomics, as well as epigenomics, proteomics and metabolomics, provides a promising route for gaining new insights into tardigrade stress tolerance and cryptobiotic survival. Specifically, combining a multi‐omics strategy with experimental approaches, such as RNAi‐mediated knockdown of single genes, followed by evaluation of the phenotypic effect, and, for example, recombinant production of the relevant protein for functional characterisation in vitro, offers the possibility of linking genotype to phenotype with the potential to discover general as well as lineage‐specific innovations. For example, Li et al. (2024) recently used multi‐omics as a launching point and further investigated molecular functions using iRNA knockdown in combination with biochemical and cell‐based assays.

It should also be noted that the use of single‐cell RNA sequencing (scRNA‐seq) has expanded rapidly over the past decade, and technological advances in single‐cell isolation and sequencing have made it possible to investigate cellular diversity in a broad range of organisms (e.g., Sebé‐Pedrós et al. 2018; Yang, Chen, et al. 2022), potentially opening new paths for investigations into tardigrade resilience. Hence, applying novel RNA‐based methods to investigate the cellular landscape of tardigrades is likely feasible within the near future. As such, future single‐cell analyses combining scRNA‐seq, for example, involving isolated tardigrade coelomocytes, with either spatial‐transcriptomics or proteomics, will allow for a far better understanding of the cellular complexities of these micro‐animals. Moreover, it has recently been suggested that injections of CRISPR genome‐editing components into mature parthenogenetic tardigrade females could be a promising method for heritable gene‐editing in tardigrades (Kumagai et al. 2022; Kondo et al. 2024), allowing for detailed investigations into the phenotypic importance of selected genes. Importantly, CRISPR‐Cas genome editing (Jinek et al. 2012) provides the possibility of simultaneous disruption of several genes, which will be necessary to understand possible epistatic relationships in the genetic pathways underlying tardigrade stress tolerance. The technique could enable the transfer of such genes to other organisms, thereby opening the opportunity for new paths in, for example, bio‐ and space‐technology.

## 6 Concluding Remarks

Recent advances in sequencing technologies, combined with experimental approaches, have reshaped our understanding of mechanisms involved in tardigrade stress tolerance. With this review we explore state‐of‐the‐art within this field and we consolidate dispersed evidence on tardigrade stress tolerance into a reusable community resource. Specifically, we provide curated gene sets for stress protection, 3D structure and peroxin modules, enabling consistent cross‐study comparisons. We further release a new transcriptome for the heterotardigrade *Echiniscus testudo* and interactive recovered/non‐recovered matrices and top‐hit tables for 15 species, which other researchers can mine directly or extend to new taxa, improving transparency and reproducibility of comparative inferences.

Based on the studies included in this review, there is no indication that the extreme stress tolerance of tardigrades relies on one single genetic factor, but rather seems to be caused by the interplay of multiple genetic pathways that may differ between tardigrade lineages. We introduce a functional presence‐score framework that standardises how multi‐gene pathways are summarised across species, facilitating meta‐analysis and hypothesis testing. Our synthesis further highlights testable predictions, such as lineage‐specific losses and gains, guiding targeted validation with HMM/domain and synteny searches. Our synthesis identifies tractable candidates and data gaps, including epigenomics, proteomics, metabolomics and single‐cell approaches, while also discussing experimental steps and methods that potentially can be used to link genotype to phenotype, including RNAi‐mediated knockdowns and CRISPR‐Cas genome editing. Specifically, multi‐omics, in combination with novel RNA‐based technologies, biochemical assays and experimental physiology, provides a promising route for obtaining further insights into the unique survival strategies of these enigmatic animals.

## 7 Methods

### 7.1 New De Novo Transcriptome Assembly of *Echiniscus testudo*

Total RNA (1.4 μg) was extracted from 525 *Echiniscus testudo* specimens collected from a roof gutter in Nivå, Denmark (55°56′36.53″ N, 12°30′00.90″ E). The RNA was sent to BGI Europe A/S, where sample quantity and quality were evaluated (Agilent 4200), followed by mRNA selection, reverse transcription and short pair‐end sequencing (100 bp) using DNBSEQ‐G400 technology. Basic bioinformatic analyses were also conducted by BGI. In brief, raw reads were quality filtered by removal of adapters, trimming of low‐quality bases and discarding of short/low‐quality reads. The cleaned reads were used to generate a de novo transcriptome assembly with Trinity v2.0.6 using ‐‐min_kmer_cov 3 to reduce assembly noise from low‐abundance k‐mers and improve contiguity. To reduce redundancy, assembled isoforms were clustered using TGICL (v2.0.6) with default parameters, retaining the longest representative sequence per cluster; this non‐redundant transcript set was treated as ‘Unigenes’ (putative gene‐level units) for downstream comparative analyses. Sequencing yield and quality metrics (raw reads, Q20/Q30 and post‐filtering read counts) are reported in Table S1. For functional annotation, Unigenes were queried against multiple reference databases, including NT, NR, GO, KOG, KEGG, SwissProt and InterProScan. Nucleotide‐level annotation against NT was performed using BLASTn. Protein‐level annotation was performed by aligning Unigene translations to NR, KOG, KEGG and SwissProt using BLASTx and/or DIAMOND. Gene Ontology (GO) terms were assigned using Blast2GO based on the NR annotation results. Protein domain and motif annotations were generated using InterProScan v5, and InterPro accessions were retained as complementary functional evidence alongside sequence‐homology‐based annotations.

### 7.2 Data Mining and Meta‐Analysis

We compiled a curated list of genes implicated in cryptobiosis, stress response, 3D structure and peroxisome biology from prior literature to investigate the recovery or non‐recovery of these genes using BLAST (Workbooks S1, S2 and S3; BLAST+ 2.6.0; Altschul 1997). Species‐specific transcriptome and genome assemblies were used to build per‐species search databases (Tables 1 and S2). Because most queries were available only as nucleotides, we used tBLASTx (six‐frame translation of query and database) to detect homologues despite uncertain ORFs and divergence. For the genes implicated in peroxisome biogenesis—for which curated protein sequences were available—we used tBLASTn (protein vs. six‐frame translated nucleotide database) to reduce spurious UTR matches and avoid query‐side frame artefacts. For both modes, we used identical reporting thresholds (*E*‐value cut‐off 1 × 10^−4^). For each species, we exported ‘All Hits’ (all alignments with *E* ≤ cut‐off) and ‘Top Hits’ (best hits per query ranked by lowest *E*‐values); ties were broken by (i) higher bit score, then (ii) percentage of identical matches and finally by (iii) percentage of query coverage. Recovery of the relevant genes was called TRUE if a specific species assembly contained ≥ 1 hit for a curated gene ID; otherwise, FALSE. This mixed tBLASTx and tBLASTn design keeps the biological signal at the amino‐acid level in all cases, while matching the search mode to the data type, thereby minimising false negatives without inflating false positives. Mode‐specific artefacts (short spurious ORFs in tBLASTx; domain‐only hits in tBLASTn) were mitigated by minimum alignment length and coverage checks, low‐complexity masking and (where needed) reciprocal search confirmation. For each search, the corresponding database matches in the species assemblies are reported under ‘Subject accession dot version (database hit)’ in the BLAST output tables.

Completeness of retrieved datasets was assessed using BUSCO v5.8.0 in transcriptome mode (‐‐mode euk_tran) against the metazoa_odb10 lineage dataset (creation date 2021‐02‐17; 65 reference genomes; 954 BUSCOs). BUSCO was executed with the bundled workflow using MetaEuk v7.bba0d80 and HMMER hmmsearch v3.4 under Python 3.10.16. For each assembly, we report BUSCO scores in standard notation (Complete [Single‐copy + Duplicated], Fragmented and Missing) as a measure of assembly completeness (Table S2). Results of our data mining and meta‐analysis are presented in Figures 3, 4 and S1.

## Author Contributions

Maria Kamilari performed the data mining and sequence analyses and prepared Figures 3 and 4 and the Supporting Information. Frida Løkkegaard Pust, Andrea Marcantognini and Ask Møbjerg prepared Table 1 and Figure 2. Ricardo Cardoso Neves prepared *Echiniscus testudo* specimens and Morten Schiøtt extracted RNA for the reference transcriptome. Nadja Møbjerg conceived the study, provided tardigrades, prepared Figure 1 and initial lists of genes and species with available assemblies, and wrote the manuscript with contributions from all authors.

## Funding

This work was supported by a research grant (17522) from Villum Fonden.

## Conflicts of Interest

The authors declare no conflicts of interest.

## Supporting information


**Figure S1:** Functional recovery scores of stress tolerance‐related genes across tardigrades. Heatmap summarising the recovery of stress‐ and adaptation‐related features in 15 tardigrade species (rows) mapped to a display‐only phylogeny (left). Cell values are ‘recovery scores,’ not gene copy numbers: for each functional category (columns), we summed binary recoveries of predefined submarkers from the detailed recovery/not recovery table (see Figure 3). Thus, higher numbers indicate more submarkers recovered for each category in a specific species. Category maxima differ; see Methods and Workbooks S1 and S3 for sub marker lists and thresholds. Shading follows the scale (0–max) shown below. Right‐hand annotation bars indicate habitat, class or order (Parachela = P, Apochela = A, Heterotardigrada), family and tun formation. Categories: Radiotolerance (DSup, TRID1, TDR1); Intrinsically Disordered Proteins (IDPs: CAHS, SAHS, MAHS, LEA, EtAHs); Trehalose metabolism (TPS‐TPP, ATHL1, TREH); Cold‐shock domain factors (CSD: YB, CSP); Heat Shock Proteins (HSPs); Antioxidants (SOD, CAT, PRDX, TXN/TXNL/TXNRD, GPX, GST, GSS, AMNP, AMNP‐like, OG0000230, CAT, DODA1); DNA repair (MRE11, XRCC, p53, RAD); Aquaporins (AQP); Cytoskeleton filaments (actin, myosin, paramyosin, troponin, tropomyosin, lamin, cytotardin, tubulin, collagen); and putative HGTs (CAT, DODA1, TPS‐TPP).

**Figure S2:** Schematic of peroxisome biogenesis and matrix import in tardigrades based on recovery/non‐recovery calls. Receptors (PEX5, PEX7), docking (PEX13/PEX14), RING E3 ligase (PEX2/PEX10/PEX12), AAA‐ATPase recycling (PEX1/PEX6 ± PEX26), membrane biogenesis (PEX3/PEX19/PEX16), division (PEX11), matrix protease (TYSND1) and transporters/channels (PMP34/SLC25A17, ABCD/PMP70, PXMP2, PXMP4) are shown. Red stars denote genes not recovered in any of the screened species; coloured tiles within each module summarise species‐level recovery/non‐recovery. Aa: *Acutuncus antarcticus* , Et: *Echiniscus testudo*, Es: *Echiniscoides sigismundi*, He: *Hypsibius exemplaris*, Hh: *Hypsibius henanensis*, Mi: *Milnesium inceptum*, Pf: *Paramacrobiotus fairbanski*, Pm: *Paramacrobiotus metropolitanus*, Pcfr: *Paramacrobiotus* cf. *richtersi*, Ps: *Paramacrobiotus spatialis*, Rc: *Richtersius coronifer s.s*, Ri: *Richtersius ingemari*, Rv: *Ramazzottius varieornatus*, Rcfv: *Ramazzottius cf. varieornatus*.

**Table S1:** Sequencing, assembly, completeness and annotation statistics for the *Echiniscus testudo* transcriptome (data from BGI).
**Table S2:** BUSCO completeness statistics for genome and transcriptome assemblies used in this study.

**Data S1:** Workbook S1: BLAST results related to the investigated stress tolerance‐related genes across currently available tardigrade assemblies. Workbook structure: Sheet 1 = Summary (interactive recovery/non‐recovery): Species are rows; genes of interest are columns. Cells display TRUE/FALSE based on whether that species has ≥ 1 top hit for the gene (pulled from each species sheet via INDIRECT+COUNTIF). Right‐side panels summarise per‐gene prevalence (%) and per‐species gene counts.; Sheets 2+: one sheet per species (TopHits): Each species sheet lists the best hit per query (ranked by lowest *E*‐values; ties broken by (i) higher bit score, then (ii) percentage of identical matches and finally by (iii) percentage of query coverage) at *E* ≤ 1*e*−4 using tBLASTx.

**Data S2:** Workbook S2: Blast results related to peroxisome‐related genes across currently available tardigrade assemblies. Workbook structure: Sheet 1 = Summary (interactive recovery/non‐recovery): Species are rows; genes of interest are columns. Cells display TRUE/FALSE based on whether that species has ≥ 1 top hit for the gene (pulled from each species sheet via INDIRECT+COUNTIF). Right‐side panels summarise per‐gene prevalence (%) and per‐species gene counts.; Sheets 2+: one sheet per species (TopHits): Each species sheet lists the best hit per query (ranked by lowest *E*‐values; ties broken by (i) higher bit score, then (ii) percentage of identical matches and finally by (iii) percentage of query coverage) at *E* ≤ 1*e*−4 using tBLASTn.

**Data S3:** Workbook S3: Curated gene‐marker catalogue used to define and investigate functional modules associated with tardigrade stress tolerance. The entries should be interpreted as query/reference marker sequences or gene identifiers, not necessarily as experimentally validated orthologues in every downstream species or transcriptome.

## Data Availability

The sequencing data generated in this study is available at GenBank, under the BioProject: PRJNA1461411. Blast results related to the investigated genes (cf. Workbook S3) across currently available tardigrade assemblies (cf. Table S2) are available through the Workbooks S1 and S2 uploaded as part of the Supporting Information.
