# Supplementary figures and images for: At the Edge of Survival: Exploring the Frontiers of Tardigrade Extreme Stress Tolerance
Source: Mol Ecol. 2026 Jul 22;35(14):e70471. doi: 10.1111/mec.70471 (PMC13389832; doi:10.1111/mec.70471)

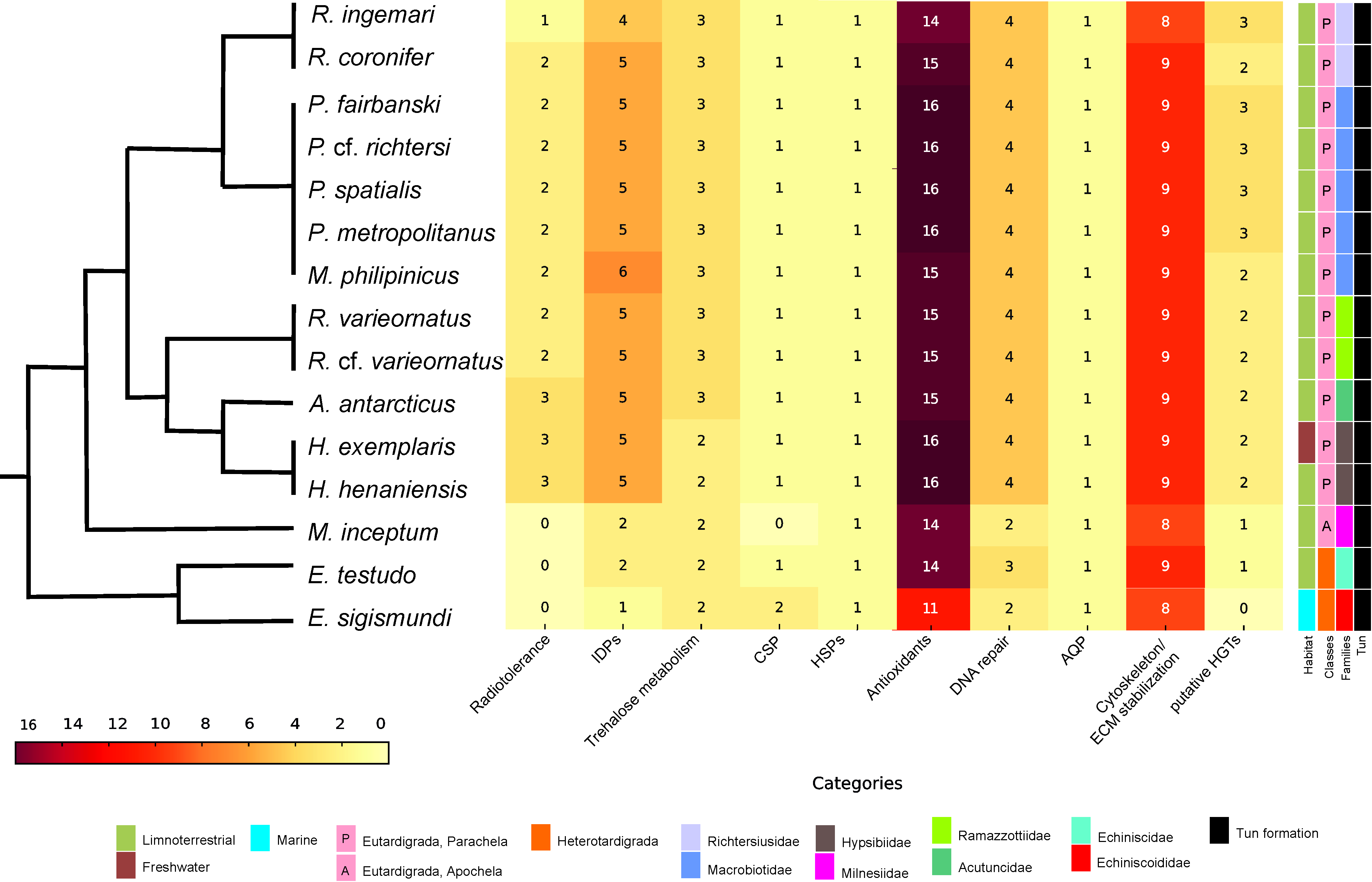

Supplement: Supplementary file 1 — Figure S1: Functional recovery scores of stress tolerance‐related genes across tardigrades. Heatmap summarising the recovery of stress‐ and adaptation‐related features in 15 tardigrade species (rows) mapped to a display‐only phylogeny (left). Cell values are ‘recovery scores,’ not gene copy numbers: for each functional category (columns), we summed binary recoveries of predefined submarkers from the detailed recovery/not recovery table (see Figure 3). Thus, higher numbers indicate more submarkers recovered for each category in a specific species. Category maxima differ; see Methods and Workbooks S1 and S3 for sub marker lists and thresholds. Shading follows the scale (0–max) shown below. Right‐hand annotation bars indicate habitat, class or order (Parachela = P, Apochela = A, Heterotardigrada), family and tun formation. Categories: Radiotolerance (DSup, TRID1, TDR1); Intrinsically Disordered Proteins (IDPs: CAHS, SAHS, MAHS, LEA, EtAHs); Trehalose metabolism (TPS‐TPP, ATHL1, TREH); Cold‐shock domain factors (CSD: YB, CSP); Heat Shock Proteins (HSPs); Antioxidants (SOD, CAT, PRDX, TXN/TXNL/TXNRD, GPX, GST, GSS, AMNP, AMNP‐like, OG0000230, CAT, DODA1); DNA repair (MRE11, XRCC, p53, RAD); Aquaporins (AQP); Cytoskeleton filaments (actin, myosin, paramyosin, troponin, tropomyosin, lamin, cytotardin, tubulin, collagen); and putative HGTs (CAT, DODA1, TPS‐TPP). [file MEC-35-e70471-s004.jpg]

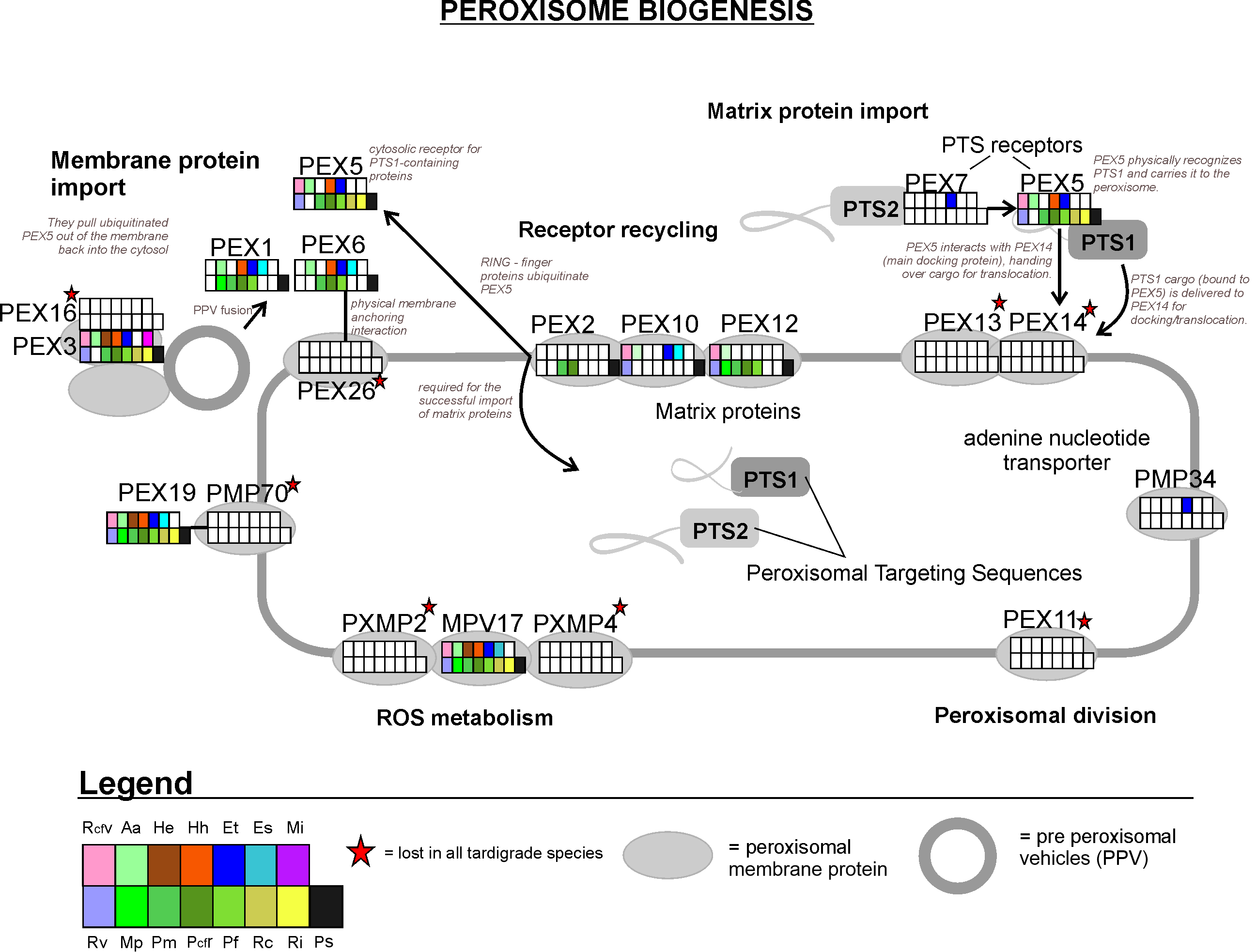

Supplement: Supplementary file 2 — Figure S2: Schematic of peroxisome biogenesis and matrix import in tardigrades based on recovery/non‐recovery calls. Receptors (PEX5, PEX7), docking (PEX13/PEX14), RING E3 ligase (PEX2/PEX10/PEX12), AAA‐ATPase recycling (PEX1/PEX6 ± PEX26), membrane biogenesis (PEX3/PEX19/PEX16), division (PEX11), matrix protease (TYSND1) and transporters/channels (PMP34/SLC25A17, ABCD/PMP70, PXMP2, PXMP4) are shown. Red stars denote genes not recovered in any of the screened species; coloured tiles within each module summarise species‐level recovery/non‐recovery. Aa: Acutuncus antarcticus , Et: Echiniscus testudo, Es: Echiniscoides sigismundi, He: Hypsibius exemplaris, Hh: Hypsibius henanensis, Mi: Milnesium inceptum, Pf: Paramacrobiotus fairbanski, Pm: Paramacrobiotus metropolitanus, Pcfr: Paramacrobiotus cf. richtersi, Ps: Paramacrobiotus spatialis, Rc: Richtersius coronifer s.s, Ri: Richtersius ingemari, Rv: Ramazzottius varieornatus, Rcfv: Ramazzottius cf. varieornatus. [file MEC-35-e70471-s005.jpg]
