# Supplementary material for: At the Edge of Survival: Exploring the Frontiers of Tardigrade Extreme Stress Tolerance
Source: Mol Ecol. 2026 Jul 22;35(14):e70471. doi: 10.1111/mec.70471 (PMC13389832; doi:10.1111/mec.70471)
Supplement: Supplementary file 3 — Table S1: Sequencing, assembly, completeness and annotation statistics for the Echiniscus testudo transcriptome (data from BGI). Table S2: BUSCO completeness statistics for genome and transcriptome assemblies used in this study. [file MEC-35-e70471-s002.docx]

**Table S1: Sequencing, assembly, completeness, and annotation statistics for the *Echiniscus testudo* transcriptome (data from BGI).**

| Category | Metric | Value |
| --- | --- | --- |
| Sequencing & QC |  |  |
|  | Clean reads Q20 | 97.49% |
|  | Clean reads Q30 | 89.47% |
|  | Clean reads ratio | 94.28% |
|  | Total clean bases | 6.62 Gb |
|  | Total clean reads | 66.17 million read pairs |
|  | Total raw reads | 70.19 million read pairs |
| Assembly (Trinity transcripts) |  |  |
|  | Mean transcript length | 823 bp |
|  | Number of transcripts | 71,883 |
|  | Total assembled transcript length | 59,187,054 bp |
|  | Transcript GC | 46.75% |
|  | Transcript N50 | 1,502 bp |
|  | Transcript N70 | 851 bp |
|  | Transcript N90 | 297 bp |
| Assembly (Unigenes; TGICL clustered) |  |  |
|  | Mean unigene length | 1,006 bp |
|  | Number of unigenes | 32,841 |
|  | Total unigene length | 33,041,571 bp |
|  | Unigene GC | 46.68% |
|  | Unigene N50 | 1,821 bp |
|  | Unigene N70 | 1,060 bp |
|  | Unigene N90 | 375 bp |
| Functional annotation (unigenes) |  |  |
|  | Annotated in GO | 5,593 (17.03%) |
|  | Annotated in InterPro | 12,095 (36.83%) |
|  | Annotated in KEGG | 11,938 (36.35%) |
|  | Annotated in KOG | 11,673 (35.54%) |
|  | Annotated in NR | 13,870 (42.23%) |
|  | Annotated in NT | 1,476 (4.49%) |
|  | Annotated in Swiss-Prot | 11,894 (36.22%) |
|  | Annotated in all 7 DBs (intersection) | 555 (1.69%) |
|  | Annotated in ≥1 DB (overall) | 15,281 (46.53%) |
| CDS prediction (TransDecoder) |  |  |
|  | CDS GC | 48.56% |
|  | CDS N50 | 1,473 bp |
|  | CDS N90 | 477 bp |
|  | Max CDS length | 10,341 bp |
|  | Min CDS length | 297 bp |
|  | Number of predicted CDS | 19,001 |
|  | Total CDS length | 20,190,879 bp |
| Read mapping to unigene reference |  |  |
|  | Mapping rate (total mapped / total reads) | 74.36% |
|  | Total mapped reads | 49,205,984 |
|  | Total reads (for mapping) | 66,173,134 |
|  | Unique mapping rate (unique / total reads) | 60.32% |
|  | Uniquely mapped reads | 39,914,028 |
| Bioinformatics pipeline (from report) |  |  |
|  | Assembly | Trinity v2.0.6; --min_contig_length 150 --CPU 8 --min_kmer_cov 3 --min_glue 3 |
|  | CDS prediction | TransDecoder v3.0.1 (default params) |
|  | Clustering/redundancy reduction | TGICL v2.0.6; -l 40 -c 10 -v 25 (incl. repeat_stringency 0.95 -minmatch 35 -minscore 35) |
|  | GO annotation | Blast2GO v2.5.0 (default params, GO from NR hits) |
|  | InterPro annotation | InterProScan5 v5.11-51.0 (default params) |
|  | NT/NR/KOG/KEGG/Swiss-Prot annotation | BLAST v2.2.23 (blastn/blastx) and/or DIAMOND v0.8.31 (default params) |

**Table S2: BUSCO completeness statistics for genome and transcriptome assemblies used in this study.**

| Species | Genome/transcriptome used_Genbank Accession number | Complete & single-copy BUSCOs (S) & duplicated BUSCOs (D) | Fragmented BUSCOs (F) | Missing BUSCOs (M) | *Completeness* |
| --- | --- | --- | --- | --- | --- |
| *Acutuncus antarcticus* | [Anoud et al. 2024_data](https://www.ncbi.nlm.nih.gov/nuccore/GKQX00000000.1?report=genbank) | C:79.9%[S:30.3%; D:49.6%] | F:4.4% | M:15.7% | ***84.3*** |
| *Ehiniscus testudo* | [Borner et al. 2014_data](https://www.ncbi.nlm.nih.gov/bioproject/236252) | C:18.4%[S:17.9%; D:0.5%] | F:10.2% | M:71.4% | ***28.6*** |
| *Ehiniscus testudo* | [Mapalo et al. 2020_data](https://dataverse.harvard.edu/dataset.xhtml?persistentId=doi:10.7910/DVN/CFNUGF) | C:67.2%[S:25.1%; D:42.1%] | F:12.3% | M:20.5% | ***79.5*** |
| *Ehiniscus testudo* | [Murai et al. 2021](https://figshare.com/articles/dataset/Assembled_Genome_and_Transcriptome_of_heterotardigrade_Echiniscus_testudo/13060634) | ***Data from Murai et. al. 2021*** | | ***after screening 87.8*** | |
| *Ehiniscus testudo* | This study/BioProject PRJNA1461411 | C:70.1%[S:56.4%,D:13.7%] | F:7.4% | M:22.4% | ***77.5*** |
| *Echiniscoides sigismundi* | [Kamilari et al. 2019](https://erda.ku.dk/public/archives/a180455075fcb29d1124fdcaf5734cf5/published-archive.html) | C:71.4%[S:64.0%; D:7.3%] | F:5.2% | M:23.4% | ***76.6*** |
| *Hypsibius henanensis* | [Li et al. 2024_data](https://download.cncb.ac.cn/gwh/Animals/Hypsibius_henanensis_Hypsibius_Henanensis_GWHDUDB00000000/) | C:81.7%[S:79.5%; D:2.2%] | F:4.9% | M:13.4% | ***86.6*** |
| *Milnesium inceptum* | [Schokraie et al. 2010/FUNCRYPTA](https://www.ncbi.nlm.nih.gov/bioproject/34121) | C:29.8%[S:29.5%; D:0.3%] | F:9.1% | M:61.1% | ***38.9*** |
| *Mesobiotus philippinicus* | [Mapalo et al. 2020](https://dataverse.harvard.edu/dataset.xhtml?persistentId=doi:10.7910/DVN/CFNUGF) | C:64.7%[S:3.4%; D:61.3%] | F:6.7% | M:28.6% | ***71.4*** |
| *Hypsibius exemplaris* | [Koutsovoulos et al. 2016](https://zenodo.org/records/45436) | C:77.9%[S:76.6%; D:1.3%] | F:8.7% | M:13.4% | ***86.6*** |
| *Hypsibius exemplaris* | [Yoshida et al. 2017](https://www.ncbi.nlm.nih.gov/datasets/genome/GCA_002082055.1/) | C:83.5%[S:80.6%; D:2.9%] | F:4.3% | M:12.2% | ***87.8*** |
| *Paramacrobiotus fairbanski* | [Anoud et al. 2024](https://www.ncbi.nlm.nih.gov/nuccore/GKQV00000000.1?report=genbank) | C:84.1%[S:14.5%; D:69.6%] | F:2.9% | M:13.0% | ***87.0*** |
| *Paramacrobiotus* cf. *richtersi* | [Hara et al. 2021](https://figshare.com/projects/Metazoan_TPS-TPP_gene_identification/36410) | C:79.4%[S:25.8%; D:53.6%] | F:7.9% | M:12.8% | ***87.3*** |
| *Paramacrobiotus* *spatialis* | [Boothby et al 2017](https://pubmed.ncbi.nlm.nih.gov/28306513/); [Giovannini et al.](https://www.nature.com/articles/s41598-022-05734-6)2022 | C:84.2%[S:15.6%,D:68.6 | F:3.5% | M:12.4% | ***87.7*** |
| *Paramacrobiotus metropolitanus* | [Hara et al. 2021](https://figshare.com/projects/Metazoan_TPS-TPP_gene_identification/36410) | C:83.0%[S:77.8%; D:5.2%] | F:4.0% | M:13.0% | ***87.0*** |
| *Richitersius coronifer s.s* | [Stec et al. 2020](https://figshare.com/articles/dataset/Transcriptome_assembly_of_Richtersius_coronifer_with_annotated_BLAST_result_against_Ramazzottius_varieornatus_/8797184) | C:53.2%[S:41.8%; D:11.4%] | F:16.0% | M:30.7% | ***69.2*** |
| *Richtersius ingemari* | [Kamilari et al. 2019](https://erda.ku.dk/public/archives/a180455075fcb29d1124fdcaf5734cf5/published-archive.html) | C:76.5%[S:59.2%; D:17.3%] | F:6.6% | M:16.9% | ***83.1*** |
| *Ramazzottius* *varieornatus* | [Neves et al. 2022](https://www.ncbi.nlm.nih.gov/Traces/wgs/HBXH01?display=download)a; [Møbjerg et al. 2022](https://pubmed.ncbi.nlm.nih.gov/35640792/) | C:84.2%[S:61.3%; D:22.9%] | F:2.8% | M:13.0% | ***87.0*** |
| *Ramazzottius* cf. *varieornatus* | [Hashimoto et al. 2016](https://www.ncbi.nlm.nih.gov/datasets/genome/?bioproject=PRJDB5011) | C:84.2%[S:79.8%; D:4.4%] | F:2.8% | M:13.0% | ***87.0*** |

For each assembly/resource (“Genome/transcriptome used”), BUSCO results are reported in standard notation as **Complete (C)** BUSCOs, partitioned into **single-copy (S)** and **duplicated (D)**, plus **Fragmented (F)** and **Missing (M)** BUSCOs. The **Completeness** column corresponds to **C + F** (i.e., the fraction of BUSCO groups recovered either completely or partially). The total number of BUSCO ortholog groups searched was n=954**.**
